# Protein Phosphorylation Dynamics Under Carbon/Nitrogen-Nutrient Stress and Identification of a Cell Death-Related Receptor-Like Kinase in Arabidopsis

**DOI:** 10.3389/fpls.2020.00377

**Published:** 2020-04-03

**Authors:** Xingwen Li, Miho Sanagi, Yu Lu, Yuko Nomura, Sara Christina Stolze, Shigetaka Yasuda, Yusuke Saijo, Waltraud X. Schulze, Regina Feil, Mark Stitt, John E. Lunn, Hirofumi Nakagami, Takeo Sato, Junji Yamaguchi

**Affiliations:** ^1^Faculty of Science and Graduate School of Life Sciences, Hokkaido University, Sapporo, Japan; ^2^Plant Proteomics Research Unit, RIKEN Center for Sustainable Resource Science, Yokohama, Japan; ^3^Max Planck Institute for Plant Breeding Research, Cologne, Germany; ^4^Graduate School of Science and Technology, Nara Institute of Science and Technology, Ikoma, Japan; ^5^Department of Plant Systems Biology, University of Hohenheim, Stuttgart, Germany; ^6^Max Planck Institute of Molecular Plant Physiology, Golm, Germany

**Keywords:** sugar, nitrogen, phosphorylation, kinase, SnRK1, receptor-like kinase, metabolism, cell death

## Abstract

Nutrient availability, in particular the availability of sugar [carbon (C)] and nitrogen (N), is important for the regulation of plant metabolism and development. In addition to independent utilization of C and N nutrients, plants sense and respond to the balance of C and N nutrients (C/N-nutrient) available to them. High C/low N-nutrient stress has been shown to arrest early post-germinative growth while promoting progression to senescence in Arabidopsis. Although several signaling components of the C/N-nutrient response have been identified, the inclusive molecular basis of plant C/N-nutrient response remains unclear. This proteome analysis evaluated phosphorylation dynamics in response to high C/low N-nutrient stress. Phosphoproteomics under conditions of C/N-nutrient stress showed a global change in the phosphorylation status of proteins, including plasma membrane H^+^-ATPase, carbon and nitrogen metabolic enzymes and signaling proteins such as protein kinases and transcription factors. Further analyses suggested that SNF1-related protein kinase 1 (SnRK1) is involved in primary C/N-nutrient signal mediation via the transcriptional regulation of C/N-regulatory kinases. We also identified a leucine-rich repeat receptor-like kinase with extracellular malectin-like domain, named as LMK1, which was shown to possess cell death induction activity in plant leaves. These results provide important insight into the C/N-nutrient signaling pathways connecting nutrition stress to various cellular and physiological processes in plants.

## Introduction

Plant growth and development are controlled by signaling pathways that are triggered by various environmental conditions and integrated with endogenous cues. Plant growth is dependent on supplies of carbon (C), in the form of sugars, and nitrogen (N) to provide energy and the major components for synthesis of structural components. It is now well-established that C and N nutrients function as signaling molecules in a wide array of cellular processes, enabling plants to coordinate their growth and development with nutrient availability (Rolland et al., 2006; Vidal and Gutiérrez, 2008). Any imbalance between C and N supplies is likely to have a detrimental effect on growth, therefore the C/N ratio in cells is very important, and plants have developed a sophisticated system to sense and respond to the ratio of available C and N nutrients (Coruzzi and Zhou, 2001; Martin et al., 2002; Sato et al., 2009). C/N-nutrient conditions can affect plant phenotypes at all stages in the plant’s life cycle. For example, the post-germination growth of Arabidopsis (*Arabidopsis thaliana*) plants is markedly inhibited in medium containing excess sugar and limiting nitrogen supplies (high C/low N-nutrient stress). Growth arrest can be lifted by either lowering the sugar concentration or increasing the nitrogen concentration, or both (Martin et al., 2002). Another example of high C/low N-nutrient stress occurs in Arabidopsis plants grown in elevated CO_2_ concentrations with limiting N, which accelerates the progression of plant senescence, including leaf yellowing and anthocyanin accumulation, during the mature developmental stage (Aoyama et al., 2014). Despite the importance of C/N-nutrient responses for proper growth and development, the underlying mechanisms remain unclear.

Previous mutant screening has led to the isolation of the ubiquitin ligase ATL31, which plays a role in the C/N-nutrient response in Arabidopsis (Sato et al., 2009). ATL31 is a member of the plant-specific RING-type ubiquitin ligase ATL family (Serrano et al., 2006; Aguilar-Hernández et al., 2011). ATL31 overexpression resulted in a phenotype insensitive to high C/low N-nutrient stress and an increase in the number of green-colored cotyledons during the early post-germinative growth stage, whereas the *atl31* loss-of-function mutant showed a hypersensitive phenotype. In mature plants, ATL31 negatively regulates the progression of leaf senescence in the presence of elevated atmospheric CO_2_ and limited N concentrations (Aoyama et al., 2014). Serine (Ser) and threonine (Thr) residues at the C-terminal region of ATL31 were shown to be phosphorylated by CBL-interacting protein kinases 7, 12, and 14 (CIPK7/12/14) (Yasuda et al., 2014, 2017). Phosphorylation of these residues was found to mediate the direct interaction with and ubiquitylation of 14-3-3 protein, resulting in proteasomal degradation of 14-3-3 under high C/low N-nutrient stress (Yasuda et al., 2014, 2017). 14-3-3 protein generally interacts with phosphorylated target proteins and regulates target functions, which modulates a wide range of physiological pathways (Comparot et al., 2003; Mackintosh, 2004; Chevalier et al., 2009; Jaspert et al., 2011). The target proteins of 14-3-3 involved in plant C/N-nutrient responses, however, remain unidentified. The phosphorylation of ATL31 by CIPK7/12/14 also increases the stability of ATL31 protein under high C/low N-nutrient stress condition (Yasuda et al., 2017). Importantly, CIPK7/12/14 are transcriptionally activated in response to high C/low N-nutrient stress, suggesting the existence of an as yet unknown upstream signaling component that mediates primary C/N-nutrient signaling in Arabidopsis plants.

In this study, we carried out phosphoproteome analysis to investigate the primary and global dynamics of C/N-nutrient related phosphorylation signals in Arabidopsis seedlings. We identified 193 proteins, the phosphorylation levels of which were responsive to short-term high C/low N-nutrient stress. Among the 193 identified phospho-regulated proteins, we found that a plasma membrane H^+^-ATPase was a C/N-responsive 14-3-3 target. Besides, we showed that SNF1-related protein kinase 1 (SnRK1), presumably regulates *CIPK7/12/14* gene expressions. We also identified a putative C/N-nutrient responsive receptor-like kinase, which possesses cell death induction activity in plant leaves. In addition, the phosphoproteomics results identified several proteins likely to modulate the progression of senescence in response to C/N-nutrient stress. These results indicate the existence of a comprehensive molecular network involved in primary C/N-nutrient signaling and metabolic adaptation.

## Materials and Methods

### Plant Materials and Growth Condition

*Arabidopsis thaliana* Columbia ecotype (Col-0) was used as the wild-type (WT) in all experiments. Transgenic Arabidopsis plants constitutively expressing FLAG-tag fused 14-3-3χ (*FLAG-14-3-3χ*) under the control of a 35S promoter in the WT background (Sato et al., 2011) and the double knockdown mutant of *SnRK1α* (*snrk1α1i/1α2*) (Sanagi et al., 2018) have been described. *Nicotiana benthamiana* plants were used for transient protein expression. Arabidopsis and *N. benthamiana* seeds were surface-sterilized and sowed on C/N-modified medium indicated in each experiment. After kept in dark at 4°C for 2–4 days to synchronize germination, the plants were grown at 22°C under short-day (8 h light/16 h dark), long-day (16 h light/8 h dark) or continuous light exposure condition as indicated in each experiment. Glucose was used as sugar and, potassium nitrate and ammonium nitrate were used as nitrogen. In most experiments, we added 100 mM Glc in the control C/N medium. To optimize the growth condition of *snrk1α1i/1α2* mutant plants, we added 10 mM Glucose in the medium for gene expression analysis using *snrk1α1i/1α2* plants.

### Phosphoproteome Analysis by LC-MS/MS

Wild-type Arabidopsis seedlings were grown in liquid MS medium containing 100 mM glucose and 30 mM nitrogen (control C/N-nutrient) for 10 days under continuous light exposure. The seedlings were transferred to control C/N-nutrient or MS medium containing 200 mM glucose and 0.3 mM nitrogen (high C/low N-nutrient) for 30 min. Phosphopeptides were enriched as described previously with minor modifications (Nakagami, 2014; Choudhary et al., 2015). LTQ-Orbitrap XL (Thermo Fisher Scientific) coupled with an EASY-nLC 1000 (Thermo Fisher Scientific) was used for nano-LC-MS/MS analyses. A self-pulled needle (150 mm length × 100 μm i.d., 6-μm opening) packed with ReproSil C18 materials (3 μm; Dr. Maisch GmbH) was used as an analytical column with a “stone-arch” frit (Ishihama et al., 2002). A spray voltage of 2,400 V was applied. The injection volume was 6 μl, and the flow rate was 500 nl min^–1^. The mobile phases consisted of 0.5% acetic acid and 2% acetonitrile (A) and 0.5% acetic acid and 80% acetonitrile (B). A three-step linear gradient of 5 to 10% B in 10 min, 10 to 40% B in 120 min, 40 to 95% B in 5 min, and 95% B for 10 min was employed. The MS scan range was m/z 300–1,400. The top 10 precursor ions were selected in the MS scan by Orbitrap with resolution = 100,000 and for subsequent MS/MS scans by ion trap in the automated gain control mode, where automated gain control values of 5.00e+05 and 2.00e+05 were set for full MS and MS/MS, respectively. The normalized collision-induced dissociation was set to 35.0. A lock mass function was used for the LTQ-Orbitrap XL to obtain constant mass accuracy during gradient analysis (Olsen et al., 2005). Multi-stage activation was enabled upon detection of a neutral loss of phosphoric acid (98.00, 49.00, or 32.66 amu) (Schroeder et al., 2004) for further ion fragmentation. Selected sequenced ions were dynamically excluded for 60 s after sequencing.

Raw data was processed using MaxQuant software (version 1.6.3.4^1^) (Cox and Mann, 2008) with label-free quantification (LFQ) and iBAQ enabled (Tyanova et al., 2016a). MS/MS spectra were searched by the Andromeda search engine against a combined database containing the sequences from Arabidopsis thaliana (TAIR10_pep_20101214^2^) and sequences of 248 common contaminant proteins and decoy sequences. Trypsin specificity was required and a maximum of two missed cleavages allowed. Minimal peptide length was set to seven amino acids. Carbamidomethylation of cysteine residues was set as a fixed modification, and phosphorylation of serine, threonine, and tyrosine residues; oxidation of methionine residues; and N-terminal acetylation of proteins allowed as variable modifications. Peptide-spectrum-matches and proteins were retained if they were below a false discovery rate of 1%. Statistical analysis of the intensity values obtained for the phosphopeptides (“modificationSpecificPeptides” output file) was carried out using Perseus (version 1.5.8.5^3^) (Tyanova et al., 2016b). Quantified peptides were filtered for reverse hits and contaminants. For further processing, only peptides containing a phospho(STY) modification were kept and the intensity values were log2 transformed. After grouping samples by condition, only those peptides were retained for the subsequent analysis that had 6 valid values in one of the conditions. Data was normalized by subtraction of the median (Matrix access = Columns, Subtract = Median). Missing values were imputed from a normal distribution using the default settings in Perseus (width = 0.3, downshift = 1.8, separately for each column). Two-sample *t*-tests were performed using a *p*-value cut-off of 0.05. The accession numbers for proteomics data generated in this study are PXD016507 for ProteomeXchange and JPST000703 for jPOST (Okuda et al., 2017). To investigate possible interactions between the C/N-responsive phosphoproteins, we used the STRING database^4^ for known and predicted protein-protein interactions with the standard setting (von Mering et al., 2005).

### Coimmunoprecipitation Analysis

Arabidopsis seedlings constitutively expressing *FLAG-14-3-3χ* were grown in liquid MS medium containing 100 mM glucose and 30 mM nitrogen (control C/N-nutrient) for 10 days under continuous light exposure. The seedlings were transferred to control C/N-nutrient or MS medium containing 200 mM glucose and 0.3 mM nitrogen (high C/low N-nutrient) for 30 min. Proteins were extracted using protein extraction buffer (50 mM Tris, 0.5% Triton X-100, 150 mM NaCl, 10% glycerol, 5 mM MgCl_2_, 1 mM EDTA, pH 7.5) supplemented with 10 μM MG132, Complete Protease Inhibitor Mixture (Roche Applied Science) and PhosSTOP phosphatase inhibitor cocktail (Roche Applied Science, Germany). Proteins were immunoprecipitated with anti-FLAG M2 magnetic beads (Sigma-Aldrich, M8823) for 1 h at 4°C with shaking. After washing the beads, the bound proteins were eluted with 150 μg/ml 3x FLAG peptide (Sigma-Aldrich, F4799), followed by precipitation in cold acetone and resuspension in SDS sample buffer (62.5 mM Tris, 2% SDS, 10% glycerol, 5% 2-mercaptoethanol, 0.01% bromophenol blue, pH 6.8). The proteins were analyzed by immunoblotting with anti-FLAG (MBL, PM020) and anti-plasma membrane H^+^-ATPase (Agrisera, AS07 260) antibodies.

### Gene Expression Analysis

WT and inducible RNAi knockdown mutant of *SnRK1α* (*snrk1α1i/1α2*) plants were grown for 11 days on medium containing 10 mM glucose and 30 mM nitrogen in the absence of dexamethasone (DEX) under 16 h light/8 h dark cycles, transferred to medium supplemented with 10 μM DEX, and grown for 5 days. Total RNA was isolated from indicated plant materials using TRIzol reagent (Invitrogen) and treated with RQ1 RNase-free DNase (Promega) according to the manufacturers’ protocols. First-strand cDNA was synthesized using oligo(dT) primer (Promega) and ReverTraAce reverse transcriptase (TOYOBO) and subjected to qRT-PCR analysis on a Mx3000P system (Agilent Technologies) using TB Green Premix EX Taq (TaKaRa) and the primers listed in Supplementary Table S1, as described by the manufacturer.

### Quantification of T6P, G6P, and UDP-Glc

WT plants were grown for 16 days on medium containing 100 mM glucose and 30 mM nitrogen (control) under 16 h light/8 h dark cycles, and transferred to control medium or modified C/N-nutrient medium containing 100 mM glucose and 0.3 mM nitrogen, 300 mM glucose and 30 mM nitrogen or 300 mM glucose and 0.3 mM nitrogen. The seedlings were harvested 1 and 24 h later. T6P, G6P, and UDP-Glc were extracted with chloroform/methanol and measured by anion-exchange high performance liquid chromatography coupled to tandem mass spectrometry (LC-MS/MS) as described in Lunn et al. (2006) with modifications as described in Figueroa et al. (2016).

### Plasmid Construction

The coding sequence of *LMK1* (*At1g07650.1*) was amplified from Col-0 cDNA. LMK1 with a point mutation (LMK1D805A) and truncated forms of LMK1 (LMK1ΔL, LMK1ΔM, and LMK1ΔLΔM) were generated by PCR-based site-directed mutagenesis using the primers listed in Supplementary Table S1. Amplified fragments were cloned into pENTR/D-TOPO vector and transferred to destination vectors using the Gateway system according to the manufacturer’s protocol (Invitrogen). The sequences of all amplified fragments and inserts were verified by DNA sequencing.

### Transient Expression in *N. benthamiana* Leaves

The surface-sterilized *N. benthamiana* seeds were sowed on 1xMurashige and Skoog (MS) medium supplemented with 1% sucrose, and grown under 16 h light/8 h dark cycles at 22°C on the plates for 2 weeks, then transferred to flowerpot with soil containing compost and vermiculate in the ratio of 1:6 and grown for additional 2 weeks. The various forms of *LMK1* were subcloned into the destination vector pMDC83 (Curtis and Grossniklaus, 2003), with all genes under the control of a CaMV35S promoter and GFP attached to the C-terminal of each encoded protein. These plasmids were introduced into *Agrobacterium tumefaciens* strain GV3101 (pMP90) by electroporation using a MicroPulser electroporator (Bio-Rad), and the subcloned sequences were transiently expressed in *N. benthamiana* as described (Yasuda et al., 2014).

### Transient Expression in Arabidopsis Mesophyll Protoplast Cells

The surface-sterilized WT Arabidopsis seeds were sowed on 1xMurashige and Skoog (MS) medium supplemented with 1% sucrose, and grown under 8 h light/16 h dark cycles at 22°C on the plates for 2 weeks, then transferred to flowerpot with soil containing compost and vermiculate in the ratio of 1:6 and grown for additional 3 weeks. Full-length *LMK1* was subcloned into the destination vector pUGW5 (Nakagawa et al., 2007), with the gene under the control of a CaMV35S promoter and GFP attached to the C-terminal of each encoded protein. The plasmids were introduced by polyethylene glycol-mediated transformation (Yoo et al., 2007) into Arabidopsis mesophyll protoplasts prepared from leaf tissues, and images were taken 16 h later.

### Confocal Laser-Scanning Microscopy

Fluorescent images were obtained using a Zeiss LSM510 confocal laser scanning microscope equipped with a C-Apochromat (×40/1.2 water immersion) objective 2 days after the inoculation of *Agrobacterium* into *N. benthamiana* leaves. GFP fluorescence was excited by a 488 nm argon laser and detected using a 505–550 nm band-pass emission filter. Images were processed using Image J software.

### Preparation of Water-Soluble and Membrane Fractions

Proteins were extracted from *N. benthamiana* leaves expressing GFP or LMK1-GFP using extraction buffer (50 mM Tris, 150 mM NaCl, 10% glycerol, 1 mM EDTA, pH 7.5) supplemented with 10 μM MG132, Complete Protease Inhibitor Mixture (Roche Applied Science) and PhosSTOP phosphatase inhibitor cocktail (Roche Applied Science, Germany). Lysates were centrifuged at 20,000 *g* for 5 min at 4°C to remove cell debris, followed by ultracentrifugation (101,000 *g*, 1 h, 4°C) to separate the soluble and insoluble fractions. Membrane fraction proteins were solubilized by the extraction buffer supplemented with 1% Triton X-100. GFP and LMK1-GFP proteins were enriched by immunoprecipitation using anti-GFP antibody beads (MBL, cat. no. D153-10), followed by SDS-PAGE and western blotting analysis with anti-GFP antibody (MBL, cat. no. 598).

### Electrolyte Leakage Analysis

Ion leakage was assayed with infiltrated *N. benthamiana* leaf discs. Forty-eighth after infiltration, 15 leaf discs, each 6 mm in diameter, were generated from four different leaves of each construct. After washing with Milli-Q water, the leaf discs were transferred to tissue culture plates. Each well-contained five leaf discs and 3 ml fresh Milli-Q water. The conductivity of the water was measured at 0, 24, 48, and 72 h using a conductivity meter (HORIBA, LAQUAtwin-EC-33).

## Results

### Phosphoproteome Analysis Reveals Protein Phosphorylation Dynamics in Response to High C/Low N-Nutrient Stress in Arabidopsis Plants

**FIGURE 1:**
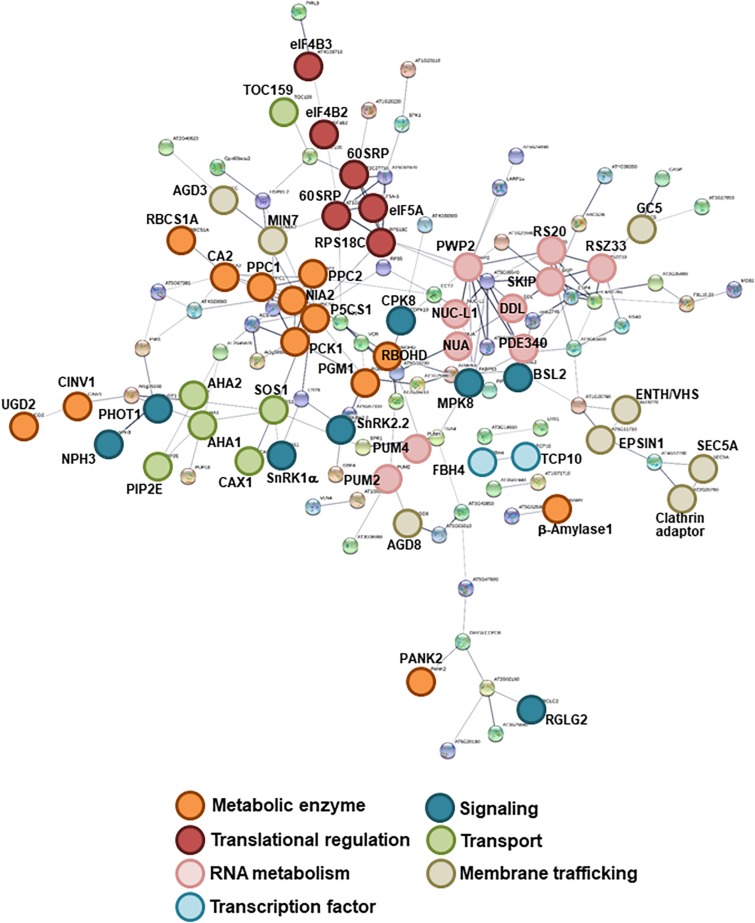
Interaction networks of the identified C/N-nutrient responsive phosphoproteins. Functional network mapping was performed using the STRING protein interaction algorithm (https://string-db.org/). Shown are the associations among C/N-nutrient responsive phosphoproteins identified by LC-MS/MS analysis (Supplementary Table S2). Nodes with connections are shown. Line thickness indicates the strength of supporting data.

To assess the effects of high C/low N-nutrient stress on global protein phosphorylation status in Arabidopsis, 10-day-old seedlings were transiently treated with control (100 mM Glc/30 mM N) or high C/low N-nutrient stress (200 mM Glc/0.3 mM N) medium for 30 min and subjected to phosphoproteome analysis with HAMMOC-TiO_2_ phosphopeptide enrichment followed by LC-MS/MS analysis. This analysis detected a total of 1785 phosphopeptides, of which 1338 were quantified, with 193 protein groups having significantly changed phosphorylation levels in response to high C/low N-nutrient stress (Table 1 and Supplementary Table S2). The functional networks of the C/N-responsive phosphoproteins were assessed using the STRING (Search Tool for the Analysis of Interacting Genes/Proteins) algorithm (Figure 1). Seven groups of proteins with distinct molecular functions were identified. Metabolic enzymes that function in photosynthesis [*rubisco small subunit 1A* (RBCS1A), carbonic anhydrase 2 (CA2), phosphoenolpyruvate carboxylases 1/2 (PPC1/2), phosphoenolpyruvate carboxykinase 1 (PCK1), cytosolic invertase 1 (CINV1), phosphoglucomutase (PGM), β-amylase1] and nitrate reductase 2 (NIA2) formed a highly connected network. Proteins involved in RNA metabolism and translational regulation also formed clusters, including RNA-binding Pumilio proteins (APUM2/4), FHA (forhkead) domain-containing protein involved in miRNA biosynthesis (DDL), pre-18S ribosomal RNA processing factor (PWP2), and 40S and 60S ribosomal subunits. Several transporters and membrane trafficking regulators were identified as being C/N-responsive phosphoproteins, including plasma membrane intrinsic protein (aquaporin) 2E (PIP2E), plasma membrane H^+^-ATPase (AHA1/2), clathrin adaptor, clathrin-interacting ENTH/VHS protein (EPSIN1), golgin candidate 5 (GC5), and brefeldin A-inhibited guanine nucleotide-exchange protein 5 (BIG5/MIN7). Besides, transcription factors TCP domain protein (TCP10) and FLOWERING bHLH (FBH4), as well as several signaling proteins, including protein kinases [SNF1-related protein kinase catalytic subunit alpha (SnRK1α), SnRK2.2, calcium-dependent protein kinase (CDPK19/CPK8), phototropin 1 (phot1)], phosphatases [BRI1 suppressor 1-like 2 (BSL2)] and ubiquitin ligase [Non-phototropic Hypocotyl 3 (NPH3), RING domain ligase 2 (RGLG2)] were identified.

**TABLE 1:** List of C/N-responsive protein kinases identified by LC-MS/MS analysis.

AGI code^a^	Protein description	Phosphopeptide^b^	Ratio (log2)^c^	*p*-Value^d^
At3g50500	SNF1-related protein kinase 2.2 (SnRK2.2)	S(0.158)S(0.172)VLHS(0.699)QPKS(0.694)T(0.25)VGT(0.026) PAYIAPEILLR	2.34	0.02
		S(0.005)S(0.005)VLHS(0.016)QPK**S**(0.812)T(0.313)VG**T**(0.787) PAY(0.06)IAPEILLR		
At1g07650	Leucine-rich repeat malectin kinase (LMK1)	S(0.002)L**S**(0.997)FSTSGPR	1.27	0.01
At3g58640	Mitogen activated protein kinase kinase kinase-like protein	RS(0.333)IS(0.333)IT(0.333)PEIGDDIVR	1.04	0.01
At3g25840	Spliceosome-associated kinase (PRP4KA)	DIVPET(0.009)GAPVS(0.455)T(0.455)S(0.081)PAVVIAANVGQAK	0.41	0.03
		DIVPET(0.001)GAPVS(0.114)T(0.114)**S**(0.772)PAVVIAANVGQAK		
		DIVPE**T**(0.751)GAPVS(0.122)T(0.122)S(0.005)PAVVIAANVGQAK		
		DIVPET(0.007)GAPVS(0.045)T(0.474)S(0.474)PAVVIAANVGQAK		
At3g17850	Incomplete root hair elongation 1 (IREH1)	VSNSHLTEESDVL**S**(1)PR	–0.46	0.01
At1g18150	Mitogen-activated protein kinase 8 (MPK8)	AAAAVASTLESEEADNGGGY**S**(1)AR	–0.56	0.01
At5g19450	Calcium-dependent protein kinase 19 (CDPK19/CPK8)	SNPFYSEAYTT(0.003)NG**S**(0.991)GT(0.006)GFK	–0.69	0.03
		SNPFYSEAYTT(0.005)NGS(0.092)G**T**(0.903)GFK		
At1g53165	AtMAP4K alpha1	DSYQNDY(0.001)QEEDDS(0.725)S(0.072)GS(0.072)GT(0.072) VVIRS(0.059)PR	–0.76	0.03
		DSYQNDY(0.007)QEEDDS(0.099)S(0.099)GS(0.094)GT(0.071) VVIRS(0.63)PR		
At3g45780	phototropin 1 (phot1/NPH1/RPT1)	AL**S**(1)ESTNLHPFMTK	–1.28	0.00
At3g01090, At3g29160	SNF1-related protein kinase 1α1, 1α2 (SnRK1α1/AKIN10, SnRK1α2/AKIN11)	DGHFLKT(0.212)S(0.212)CGS(0.576)PNYAAPEVISGK	–2.39	0.01

*^a^Accession numbers of Arabidopsis genes. ^b^Phosphopeptide sequences were detected by LC-MS/MS; phosphorylated amino acids with the localization probability score and are underlined and in bold type when the score is higher than 0.75. ^c^Average log2 fold difference between high C/low N-nutrient stress and control conditions. ^d^Calculated by Student’s t-tests.*

### Physical Interaction Between H^+^-ATPase and 14-3-3 Is Enhanced Under High C/Low N-Nutrient Stress Conditions

**FIGURE 2:**
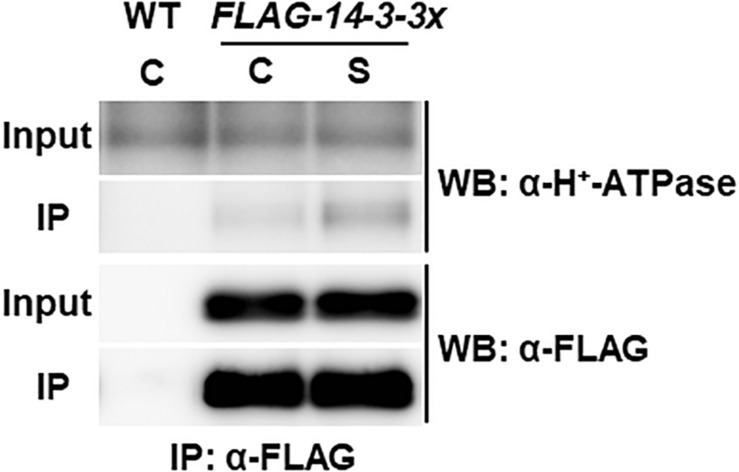
Co-immunoprecipitation analysis of plasma membrane H^+^-ATPase and 14-3-3 proteins. WT and transgenic Arabidopsis plants expressing FLAG-14-3-3χ were grown in control liquid medium containing 100 mM Glc/30 mM N, and 10-day-old seedlings were treated with the control (C) or the high C/low N-nutrient stress medium containing 200 mM Glc/0.3 mM N (S) for 30 min. Proteins were extracted and subjected to immunoprecipitation with anti-FLAG antibody beads, followed by immunoblotting with anti-plasma membrane H^+^-ATPase and anti-FLAG antibodies. Three independent experiments showed similar results.

Plasma membrane H^+^-ATPases act as primary transporters of H^+^, which regulates pH homeostasis and membrane potential and drives several nutrient transport processes. High C/low N-nutrient stress increased the phosphorylation levels of Thr residues at the C-terminal regions of plasma membrane H^+^-ATPases 1 and 2 (AHA1 and AHA2; Figure 1 and Supplementary Table S2). Phosphorylation of the Thr948 residue in AHA1 and the Thr947 residue in AHA2 has been reported to mediate the binding of 14-3-3 protein, which promotes the activity of H^+^-ATPases (Kinoshita and Shimazaki, 1999; Svennelid et al., 1999; Palmgren, 2001; Chevalier et al., 2009). Therefore, we asked if there is any link between high C/low N-nutrient stress response and AHA–14-3-3 interaction. The transgenic Arabidopsis seedlings constitutively expressing FLAG-tag fused 14-3-3χ (*FLAG-14-3-3χ*) were transiently exposed to high C/low N-nutrient stress, and then the interaction was evaluated by co-immunoprecipitation. As shown in Figure 2, AHA protein abundance was not affected after the stress exposure but enhanced interaction between AHA and FLAG-14-3-3χ was observed under high C/low N-nutrient stress conditions. These results suggest that C/N conditions modulate H^+^-ATPase activity via 14-3-3 binding in Arabidopsis.

### SnRK1 Is Involved in the Transcriptional Regulation of C/N-Related Protein Kinases CIPK7/12/14

**FIGURE 3:**
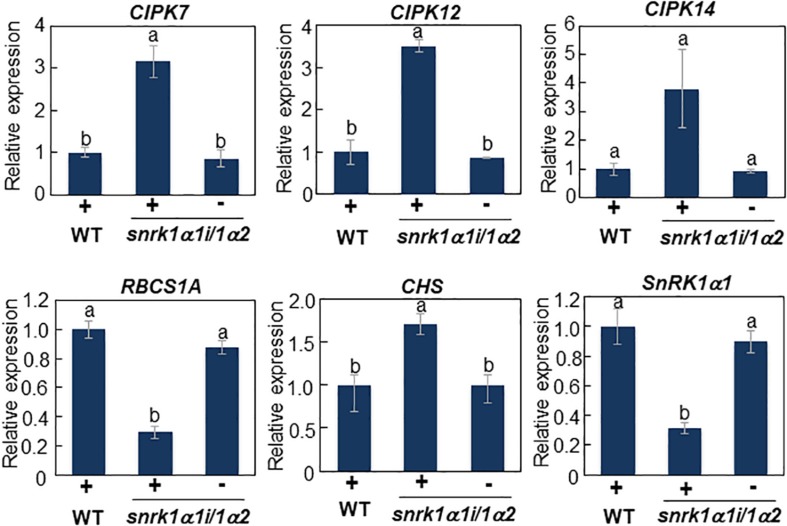
Transcript analysis of *CIPK7*, *CIPK12*, *CIPK14*, and C/N response marker genes in *snrk1α1i/1α2* mutant. WT and inducible RNAi knockdown mutant of SnRK1α (*snrk1α1i/1α2*) plants were grown for 11 days on medium containing 10 mM Glc and 30 mM N in the absence of dexamethasone (DEX) and transferred to medium supplemented with 10 μM DEX. After 5 days, total RNA was purified from each plant. Expression levels of *CIPK7/1/14* and C/N response marker genes were analyzed by qRT-PCR and normalized relative to the level of 18S rRNA in the same samples, and the expression in mutant plants was compared with that in WT plants grown in DEX-treated medium. The results shown are the means ± SD of three biological replicates. Letters above the bars indicate significant differences, as assessed by one-way ANOVA with Turkey’s *post hoc* test.

We previously demonstrated that CIPK7/12/14 kinases directly phosphorylate ATL31 and play important roles in modulating plant growth in response to C/N-nutrient conditions (Yasuda et al., 2017). The expression of *CIPK* genes was increased under high C/low N-nutrient stress, with these protein products positively regulating the ability of ATL31 to adapt to these conditions, suggesting that upstream components regulate the C/N-responsive expression of *CIPK* genes. Phosphoproteome analysis showed that high C/low N-nutrient stress significantly reduced the phosphorylation level of SNF1-related kinase 1 (SnRK1) (Table 1). The putative phosphorylation sites of SnRK1 are found to be located in the activation loop conserved in AMPK-family kinases, suggesting that SnRK1 kinase activity can be affected by high C/low N-nutrient stress. This is in line with our previous reports, showing that high C/low N-nutrient stress reduced the expression of marker genes of the SnRK1 pathway (Lu et al., 2015). Taken together, these findings raised the hypothesis that SnRK1 is involved in primary signal transduction of C/N-nutrient availability. To clarify the function of SnRK1, the *CIPK7/12/14* gene expression levels were measured in *SnRK1α* knockdown plant (*snrk1α1i/1α2*), inducible RNAi knockdown of *SnRK1α1* in the background of a *SnRK1α2* knockout mutant (Sanagi et al., 2018). Induced *SnRK1α1* gene silencing upon DEX treatment resulted in elevated expression of *CIPK7/12/14* genes, suggesting that SnRK1 negatively regulates *CIPK7/12/14* gene expression (Figure 3). We also measured expression levels of C/N-nutrient responsive maker genes, *RUBISCO SMALL SUBUNIT 1A* (*RBCS1A*), a photosynthesis-related gene, and *CHALCONE SYNTHASE* (*CHS*), a key enzyme that regulates anthocyanin biosynthesis. *RBCS1A* gene expression was significantly decreased and *CHS* expression was significantly increased in the *snrk1α1i/1α2* mutant compared with WT (Figure 3), an expression pattern similar to that observed in WT plants grown under high C/low N-nutrient stress conditions (Martin et al., 2002; Sato et al., 2009; Aoyama et al., 2014). These results suggest that SnRK1 functions as an upstream signaling component in plant C/N-nutrient response.

### Trehalose 6-Phosphate Amounts Are Responsive to C/N-Nutrient Availability

**FIGURE 4:**
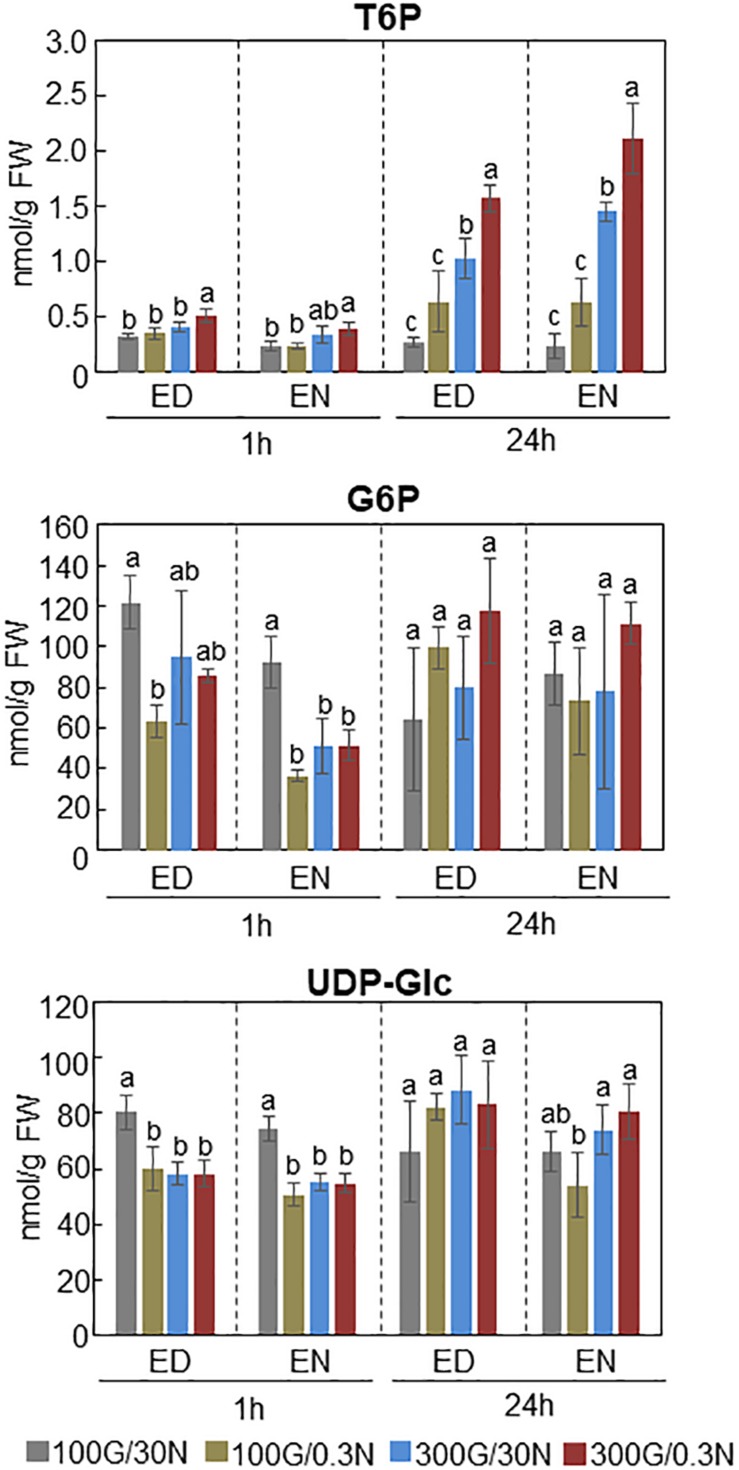
Amounts of T6P, G6P, and UDP-Glc present in WT plants grown under different C/N-nutrient conditions. WT plants were grown for 16 days on medium containing 100 mM Glc and 30 mM N (control), and transferred to control medium or modified C/N-nutrient medium containing 100 mM Glc and 0.3 mM N, 300 mM Glc and 30 mM N or 300 mM Glc and 0.3 mM N. The seedlings were harvested 1 h [end of day (ED) or end of night (EN)] and 24 h (ED or EN) after treatment, followed by metabolite quantification by LC-MS/MS. The results shown are the means ± SD of four biological replicates. Letters above the bars indicate significant differences, as assessed by one-way ANOVA with Turkey’s *post hoc* test.

Recent studies revealed that SnRK1 could directly bind to trehalose 6-phosphate (T6P), resulting in downregulation of the SnRK1 kinase activity in Arabidopsis (Zhang et al., 2009; Zhai et al., 2018). T6P, an intermediate in trehalose biosynthesis, is an essential signal metabolite in plants, modulating not only carbon metabolism but developmental processes such as flowering and senescence (Paul et al., 2008; Wingler et al., 2012; Wahl et al., 2013; Figueroa and Lunn, 2016). These findings prompted us to hypothesize that T6P level is affected by C/N-nutrient availability. Quantification of T6P amounts in Arabidopsis seedlings exposed to various C/N-nutrient conditions showed that cellular T6P level was increased in response to both high C and low N conditions and was synergistically increased by combined high C/low N treatment (Figure 4). T6P level was slightly but significantly higher in seedlings exposed to high C/low N (300 mM Glc/0.3 mM N) than to control (100 mM Glc/30 mM N) conditions after 1 h and apparently increased after 24 h, about sixfold at end of day and ninefold at end of night, respectively. Interestingly, the amounts of glucose 6-phosphate (G6P) and UDP-glucose (UDP-Glc), the substrates for T6P biosynthesis, did not correlate with C/N-nutrient availability and were not increased in plants exposed to high C/low N-nutrient stress (Figure 4). These results suggest that T6P is involved in the progression of C/N-nutrient responsive senescence via SnRK1.

### Identification of a Leucine Rich-Repeat Receptor-Like Kinase LMK1 as a C/N-Responsive Protein

**FIGURE 5:**
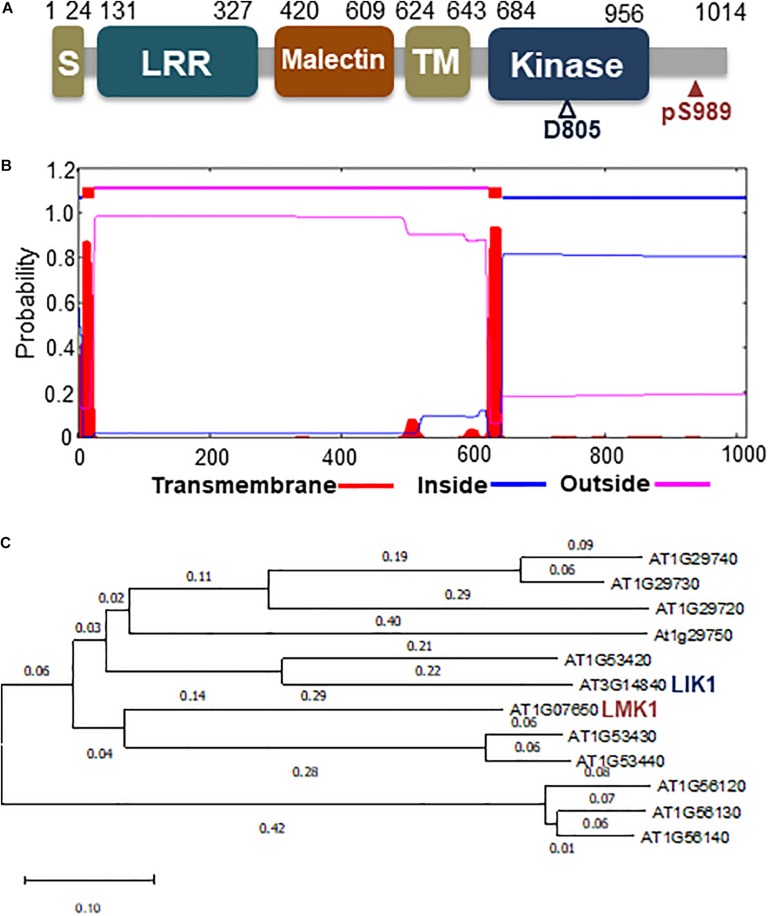
Schematic diagram of LMK1 protein and phylogenetic tree of the LRR-RLK class VIII-2 subfamily**. (A)** Schematic representation of LMK1 protein. S, signal peptide; LRR, leucin-rich repeat domain; malectin, malectin-like domain; TM, transmembrane-like hydrophobic region; kinase, cytosolic Ser/Thr kinase domain. **(B)** Predicted transmembrane region and topology of LMK1, as determined with the TMHMM server v. 2.0 (http://www.cbs.dtu.dk/services/TMHMM/). **(C)** Phylogenetic tree of LRR-RLK class VIII proteins constructed using MEGA X software with the neighbor-joining method.

Phosphoproteome analysis also showed that high C/low N-nutrient stress affected phosphorylation levels of several other protein kinases (Table 1), including At1g07650, a protein belonging to the leucine-rich repeat receptor-like kinase (LRR-RLK) family (Figure 5). LC-MS/MS analysis showed that the Ser989 residue of At1g07650 (At1g07650.1), located in the cytosolic region and close to the predicted kinase domain, was phosphorylated, with high C/low N-nutrient stress inducing a 2.4-fold higher level of phosphorylation than under control conditions (Table 1). The LRR-RLK family is one of the largest protein kinase family in plants, with over 200 members present in the Arabidopsis genome (Shiu and Bleecker, 2003). LRR-RLKs, which consist of a ligand-binding extracellular LRR domain and a cytoplasmic serine/threonine kinase domain, play critical roles in various cellular processes, directly modulating growth and development, as well as responding to environmental stress (Diévart and Clark, 2004; Macho and Zipfel, 2014). At1g07650 encodes a protein consisting of 1,014 amino acids, including LRR and Ser/Thr kinase domains, as well as an additional extracellular malectin-like domain and a transmembrane-like hydrophobic domain. This protein, named as leucine-rich repeat malectin kinase 1 (LMK1), along with 11 other proteins, formed the LRR-VIII 2 subfamily (Shiu and Bleecker, 2003) (Figure 5). The functions of these proteins remain poorly understood, although one member of this subfamily, At3g14840, also known as LysM RLK1-interacting kinase 1 (LIK1), has been reported to regulate plant innate immunity (Le et al., 2014). Amino acid sequence alignment showed that the kinase domain is highly conserved in this subfamily (Supplementary Figure S1).

### LMK1 Localizes to Membrane Compartments in Plant Cells

**FIGURE 6:**
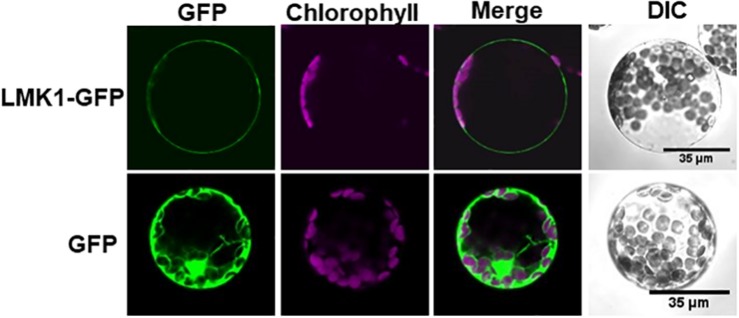
Subcellular localization of LMK1 protein. Confocal laser microscopy showing the subcellular localization of LMK1-GFP transiently expressed in Arabidopsis mesophyll protoplast cells. GFP was the control for fluorescent protein. Confocal microscopic images were taken 16 h after transfection. GFP fluorescence (GFP), chlorophyll autofluorescence (Chlorophyll) and the merged images of these fluorescence signals (Merge) were shown. DIC, differential interference contrast image. We observed more than 100 protoplast cells for each experiment and representative images are shown.

To determine the function of LMK1, we first assessed its subcellular location. Hydropathy plot analysis indicated that LMK1 has two hydrophobic regions, amino acids 7–24 and 624–643 (Figure 5B). Database search predicted that the first hydrophobic region corresponds to a signal peptide sequence (Supplementary Figure S1), suggesting that LMK1 localizes to membrane compartments. To confirm its subcellular location, LMK1 was fused to green fluorescent protein (LMK1-GFP) and transiently expressed in Arabidopsis protoplast cells and *N. benthamiana* leaves under the control of the CaMV35S promoter. Confocal microscope analysis showed that LMK1-GFP fluorescence signals were mainly detected at the periphery of the cells and partly in cytosolic dot-like structures, whereas GFP alone was present throughout cells, including the cytosol and nucleus, in both Arabidopsis protoplast cells and *N. benthamiana* leaves (Figure 6 and Supplementary Figure S2). In addition, fractionation analysis with ultracentrifuge followed by immunoblotting detected LMK-GFP signal in membrane fraction (Supplementary Figure S2), together suggesting that LMK1 localizes to the membrane compartments in plant cells.

### Overexpression of LMK1 Induces Cell Death in *N. benthamiana* Leaves

**FIGURE 7:**
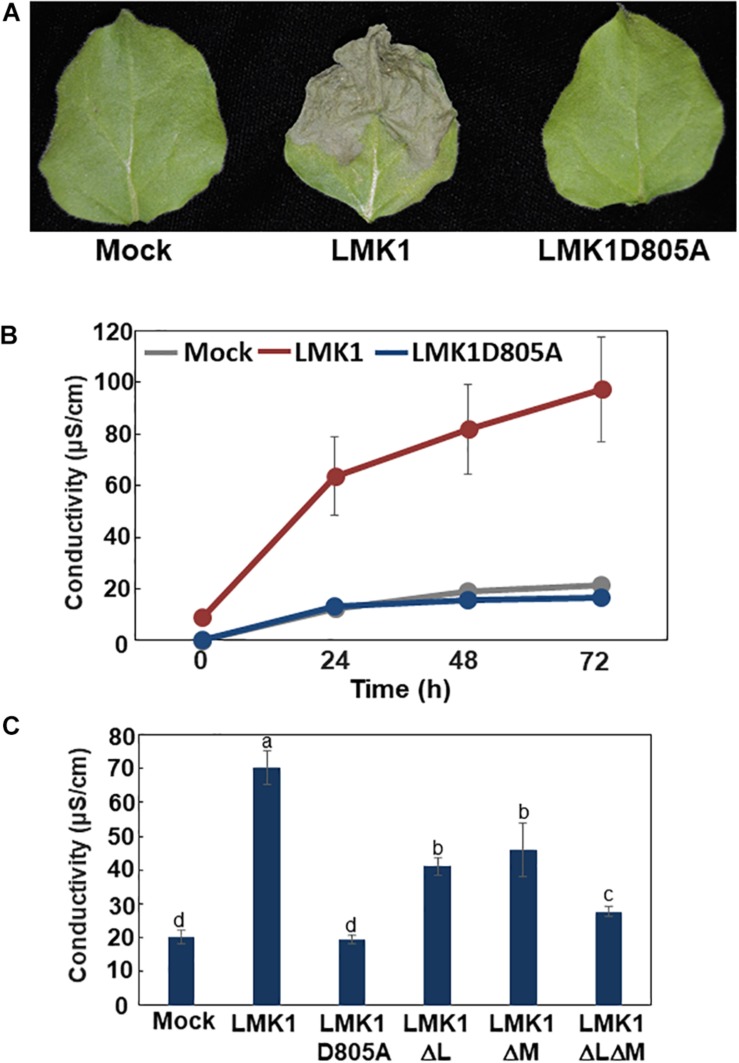
Cell death induction activity of LMK1. **(A,B)** LMK1-GFP and LMK1D805A-GFP were transiently overexpressed in *N. benthamiana* leaves. **(A)** Pictures Staken 6 days after infiltration, **(B)** Ion leakage 0, 24, 48, and 72 h after the leaf discs preparation. The results shown are the means ± SD of three biological replicates. Mock, mock treatment by infection of *Agrobacterium* carrying the p19 vector alone. **(C)** Ion leakage of *N. benthamiana* leaves transiently overexpressing LMK1-GFP and mutated LMK1 proteins fused with GFP. Ion leakage at 72 h after the cut is shown. The results shown are the means ± SD of three biological replicates. Letters above the bars indicate significant differences, as assessed by one-way ANOVA with Turkey’s *post hoc* test. Mock, mock treatment by infection of *Agrobacterium* carrying the p19 vector alone.

Transient expression of LMK1-GFP in *N. benthamiana* leaves resulted in macroscopic tissue collapse and electrolyte leakage indicative of programmed cell death (Figures 7A,B). In contrast, mock treatment by infection of *Agrobacterium* carrying the p19 vector alone showed no indications of cell death. LIK1, a homolog of LMK1, was shown to have kinase activity which requires conserved aspartate (Asp) residue in the kinase catalytic domain (Le et al., 2014) (Supplementary Figure S1). Therefore, we asked if the mutation in the Asp residue of LMK1 (LMK1D805A-GFP) alters the cell death-induction activity. Infiltration of A*grobacterium* carrying a plasmid expressing LMK1D805A-GFP did not induce cell death (Figures 7A,B and Supplementary Figure S3), implying that the observed cell death in *N. benthamiana* leaves is likely to be associated with LMK1 kinase activity. LMK1 has extracellular LRR and malectin-like domains, which are often responsible for ligand binding and protein-protein interactions. To determine roles of these extracellular domains on the cell death induction activity, the LRR domain (LMK1ΔL), the malectin-like domain (LMK1ΔM), or the both domains (LMK1ΔLΔM) were deleted and expressed in *N. benthamiana* leaves. Deletion of these domains resulted in reduction of the cell death induction activity, and deletion of both the LRR and malectin-like domains showed a synergistic effect (Figure 7C and Supplementary Figure S3). These findings suggest that the LRR and malectin-like domains of LMK1, along with the cytosolic kinase domain, are involved in the cell death induction.

## Discussion

C/N-nutrient availability has a lifelong effect on plant growth, including during early post-germinative growth and progression of senescence. Our previous study showed that high C/low N-nutrient status, resulting from elevated atmospheric CO_2_ concentration and limited nitrogen availability, promoted leaf senescence (Aoyama et al., 2014). We also identified several components mediating C/N-nutrient signaling and revealed that altered protein phosphorylation is strongly involved in responses to plant C/N-nutrient imbalance (Maekawa et al., 2012; Yasuda et al., 2014, 2017; Lu et al., 2015; Huarancca Reyes et al., 2015; Huarancca Reyes et al., 2018). However, comprehensive phosphorylation dynamics responsive to C/N-nutrient availability, including proteins targeted by 14-3-3, were unclear. The phosphoproteome analysis in the present study showed that the phosphorylation status of around 200 proteins was altered in response to high C/low N-nutrient stress. Among the proteins altered were key enzymes involved in primary carbon and nitrogen metabolism, several proteins involved in RNA metabolism and translational regulation, transporters and membrane trafficking regulators, and cellular signal transduction proteins, including protein kinases, phosphatases, ubiquitin ligases, and transcription factors.

### Plasma Membrane H^+^-ATPase

Using this approach, we found that plasma membrane H^+^-ATPases were C/N-responsive 14-3-3 target. Plasma membrane H^+^-ATPases are essential for plant life and are required for various physiological processes, including stomatal opening, nutrient uptake in roots, phloem loading, and cell expansion (Kinoshita and Shimazaki, 1999; Svennelid et al., 1999; Palmgren, 2001). Of the 11 plasma membrane H^+^-ATPases in Arabidopsis, two, AHA1 and AHA2, are the most highly expressed in seedlings and adult plants (Haruta et al., 2010). Phosphorylation has been shown to modulate plasma membrane H^+^-ATPase activities in guard cells. The Thr residues conserved in the C-termini of AHA proteins (Thr948 of AHA1 and Thr947 of AHA2) are phosphorylated in response to light stimuli, with these phosphorylated amino acids mediating direct binding to 14-3-3 and enhancing AHA activity (Kinoshita and Shimazaki, 1999; Svennelid et al., 1999; Palmgren, 2001; Chevalier et al., 2009). Phosphoproteome analysis in the present study showed that high C/low N-nutrient conditions upregulated the levels of phosphorylation of these Thr residues in AHA1 and AHA2 and enhanced the interactions between these plasma membrane H^+^-ATPases and 14-3-3 proteins, suggesting that high C/low N-nutrient conditions enhance plasma membrane H^+^-ATPase activity, at least in the short term, in Arabidopsis seedlings. Accordingly, the photosynthetic production of sugar was found to induce the phosphorylation of C-terminal Thr residues in plasma membrane H^+^-ATPases and to increase the activity of these enzymes in Arabidopsis mesophyll cells (Okumura et al., 2016).

### C/N-Related Metabolic Enzymes

Phosphoproteome analysis also revealed the phosphorylation dynamics of several enzymes catalyzing key steps of photosynthesis and primary carbon and nitrogen metabolism, including *rubisco small subunit 1A* (RBCS1A), carbonic anhydrase 2 (CA2), phosphoenolpyruvate carboxylase 1/2 (PPC1/2), phosphoenolpyruvate carboxykinase 1 (PCK1), cytosolic invertase 1 (CINV1), phosphoglucomutase (PGM), β-amylase1), and nitrate reductase (NIA2). Phosphoenolpyruvate carboxylase (PEPC) is crucial in primary metabolism, irreversibly catalyzing the conversion of phosphoenolpyruvate (PEP) to oxaloacetate (OAA) and inorganic phosphate (Chollet et al., 1996). PEPC plays central roles in glycolysis, respiration and photosynthate partitioning, as well as being an important route for carbon dioxide fixation, particularly in C4 plants. PEPC is also involved in interactions between carbon and nitrogen metabolism thorough OAA production. OAA and 2-oxoglutarate are obligate carbon skeletons that bind NH_4_^+^ and export it to other tissues (O’Leary et al., 2011). The Arabidopsis genome encodes three plant-type PEPCs, AtPPC1, AtPPC2 and AtPPC3, and one bacterial type PEPC, AtPPC4 (Shi et al., 2015). Compared with WT, a double mutant, *ppc1/ppc2*, showed altered amounts of primary carbon and nitrogen metabolites and exhibited growth arrest (Shi et al., 2015). PEPC is also suggested to be involved in carbon metabolism and amino acid remobilization during leaf senescence (Taylor et al., 2010). Due to the irreversible nature of the metabolic reaction catalyzed by PEPC, its activity is strictly regulated at the post-translational level by multiple mechanisms, including allosteric regulation and protein phosphorylation and ubiquitylation (Izui et al., 2004; O’Leary et al., 2011). Plant PEPCs are regulated by phosphorylation of the highly conserved Ser11 residue located near the N termini of PPC1 and PPC2. Phosphorylation of this residue activates PEPC by reducing its sensitivity to allosteric inhibitors while increasing its affinity for PEP (Izui et al., 2004; O’Leary et al., 2011). Our phosphoproteome analysis showed increased phosphorylation of the conserved Ser11 residues in both PPC1 and PPC2, suggesting that PEPC activity is post-translationally promoted in response to high C/low N-nutrient stress.

### Senescence-Related Proteins

Phosphoproteome analysis identified several proteins involved in leaf senescence, such as the autophagy related protein ATG13a, the reactive oxygen species (ROS)-producing enzyme RbohD and the transcription factor TCP10. Autophagy is a highly conserved cellular process in all eukaryotes, involving the degradation of intracellular constituents, making autophagy important for recycling essential nutrients under nutrient starvation conditions and during the progression of senescence. The autophagy-related protein ATG13 is involved in regulating the initiation of autophagy. In yeast, ATG13 is an accessory subunit of the ATG1/ATG13 kinase complex and positively regulates ATG1 kinase activity (Mizushima, 2010; Suzuki and Ohsumi, 2010). ATG13 is hyperphosphorylated under nutrient-rich conditions, reducing its affinity for ATG1, but is rapidly dephosphorylated under starvation conditions, resulting in increased ATG1 kinase activity and initiation of autophagy (Mizushima, 2010; Suzuki and Ohsumi, 2010; Alers et al., 2014). In Arabidopsis, ATG13 is encoded by two homologous genes, *ATG13a* and *ATG13b*, with the levels of expression of both being induced during leaf senescence (Suttangkakul et al., 2011). Moreover, double mutant of *ATG13a/b* showed accelerated senescence and was hypersensitive to nutrient limiting conditions (Suttangkakul et al., 2011). Intriguingly, levels of ATG13 phosphorylation were altered in response to sugar and nitrogen availability, although the phosphorylated sites were unknown (Suttangkakul et al., 2011). The phosphoproteome analysis in the present study consistently showed that ATG13 phosphorylation level was reduced in response to high C/low N-nutrient conditions, with Ser248 identified as a C/N-responsive phosphorylation site, suggesting that autophagy is activated via ATG13 dephosphorylation and affects senescence progression under high C/low N-nutrient stress condition.

Respiratory burst oxidase homolog (Rboh) proteins are key enzymes involved in ROS production and function in various physiological processes (Torres and Dangl, 2005). The level of RbohD phosphorylation is upregulated in response to high C/low N-nutrient conditions. Arabidopsis RbohD plays essential roles in ABA-mediated ROS production and stomatal closure (Kwak et al., 2003) and in the ROS burst of plant defense responses to pathogen attacks (Torres et al., 2002; Kadota et al., 2014). RbohD has also been reported involved in ROS production during the process of leaf senescence (Dai et al., 2018; Yang et al., 2018). Together, these results suggest that RbohD phosphorylation mediates ROS production in response to C/N-nutrient availability and may regulate plant growth pathways, including the progression of senescence and responses to biotic stress.

TCP10 is a member of transcription factor Teosinte branched 1/Cycloidea/PCF (TCP) family. TCP10 and other class II TCPs affect cell division activity and regulate leaf development under control of microRNA miR319 (Palatnik et al., 2003; Koyama et al., 2007; Nicolas and Cubas, 2016). These TCPs have also been reported to regulate the progression of senescence by promoting jasmonate biosynthesis (Schommer et al., 2008). A *jaw-D* mutant, in which levels of TCP2, TCP3, TCP4, TCP10, and TCP24 mRNAs were all strongly reduced, exhibited delayed senescence phenotype, which phenotype was restored by exogenous methyl jasmonate (Schommer et al., 2008). Although expression of these class II TCPs is tightly regulated at the post-transcriptional level by the microRNA, post-translational regulation of the TCP functions remains unclear. We found that the Thr110 residue of Arabidopsis TCP10 is phosphorylated and that its level of phosphorylation was reduced in response to high C/low-N nutrient stress, suggesting an as yet unidentified regulatory mechanism of senescence progression via TCP phosphorylation in response to C/N-nutrient status.

### Involvement of T6P-SnRK1 Module in C/N-Nutrient Signal Transduction

SnRK1 kinase, a kinase homologous to yeast sucrose non-fermenting 1 (SNF1) and mammalian AMP-activated kinase (AMPK), is a central regulator of metabolism under conditions of energy limitation and carbon starvation in plants (Baena-González et al., 2007; Smeekens et al., 2010; Emanuelle et al., 2016; Baena-González and Hanson, 2017). In addition, SnRK1 affects plant developmental processes and regulates age-dependent and dark-induced senescence progressions (Baena-González et al., 2007; Cho et al., 2012; Mair et al., 2015; Baena-González and Hanson, 2017; Pedrotti et al., 2018). The proteins SNF1, AMPK, and SnRK1 kinases form heterotrimeric holoenzymes containing a catalytic α-subunit and non-catalytic β- and γ-subunits (the latter being replaced by a hybrid bg subunit in SnRK1) (Emanuelle et al., 2016; Ramon et al., 2019). In Arabidopsis, the *AKIN10* (*SnRK1α1*) and *AKIN11* (*SnRK1α2*) genes encode the catalytic α-subunits, and plants with double knockdown of *AKIN10* and *AKIN11* showed accelerated senescence (Baena-González et al., 2007). Phosphoproteome analysis in the present study found that high C/low N-nutrient stress markedly reduced the levels of phosphorylation of the Ser/Thr residues conserved in the activation loop of AKIN10 and AKIN11. Subsequent qRT-PCR analysis demonstrated that the expression levels of *CIPK7/12/14* genes were increased in the *snrk1α1i/1α2* mutants, even under normal C/N-nutrient conditions, similar to the expression pattern found in WT seedlings grown under high C/low N-nutrient stress conditions and suggesting that SnRK1 mediates C/N-nutrient signaling upstream of CIPK7/12/14. In addition, we found that high C/low N-nutrient stress conditions increase the levels of T6P which has been implicated as a signaling metabolite that regulates progression of senescence and modulates SnRK1 activity in plants (Zhang et al., 2009; Wingler et al., 2012; Figueroa and Lunn, 2016). Recent study has demonstrated that T6P directly binds to the SnRK1 α-subunit AKIN10/11, weakening the affinity of the latter for Geminivirus rep-interacting kinases 1/2 (GRIK1/2) which phosphorylate and activate SnRK1 activity, thereby inhibiting SnRK1 activity in Arabidopsis plants (Zhai et al., 2018). Together, these results suggest that, under high C/low N-nutrient stress conditions, the T6P-SnRK1 module mediates C/N-nutrient signaling and regulates the progression of senescence via *CIPK7/12/14* transcription.

### Isolation of Cell Death Related Receptor-Like Kinase LMK1

This study found that the LMK1 protein was a putative C/N-nutrient related protein kinase belonging to the LRR-RLK class VIII-2 subfamily. LRR-RLK proteins have been reported to play essential roles in various types of cellular signaling, including phytohormone perception, pattern triggered immunity (PTI) and fertilization (Diévart and Clark, 2004; Macho and Zipfel, 2014). We found that transient overexpression of *LMK1* induces cell death in *N. benthamiana* leaves. Induction of cell death is an important process in plant defense against pathogens as well as in the progression of senescence (Lim et al., 2007; Coll et al., 2011; Mukhtar et al., 2016). Although little is known about the function of LRR-RLK class VIII-2 proteins, one of these proteins, LIK1, has been reported to interact directly with and be phosphorylated by Chitin elicitor receptor kinase 1 (CERK1) and to negatively regulate ROS production and chitin-induced immunity (Le et al., 2014).

LMK1 contains expected extracellular LRR and malectin-like domains. Transient expression of LMK1 demonstrated that both of these extracellular domains are involved in LMK1-induced cell death. Malectin protein was first identified as a carbohydrate binding protein localizing to the endoplasmic reticulum and being involved in N-glycosylation of membrane proteins in mammals (Schallus et al., 2008). Although proteins homologous to malectin have not yet been identified in plants, malectin-like domains have been detected in the extracellular regions of several receptor-like kinases. Feronia (FER) is a receptor-like kinase with two malectin-like extracellular domains that plays critical roles in controlling Arabidopsis growth, including in fertilization, senescence progression, pathogen resistance, phytohormone signaling, and starch accumulation (Haruta et al., 2014; Du et al., 2016; Liao et al., 2017; Stegmann et al., 2017; Feng et al., 2018). FER has been shown to bind via its extracellular malectin-like domains to the peptide ligand Rapid alkalinization factor (RLAF), to leucine-rich repeat extension (LRX) protein, and to cell wall pectin (Stegmann et al., 2017; Feng et al., 2018; Dünser et al., 2019). Recently, FER was reported to directly phosphorylate ATL6, the closest homolog of ATL31, and to modulate C/N-nutrient responses (Xu et al., 2019), suggesting that the extracellular malectin-like domains of FER can recognize ligands mediating C/N-nutrient availability. Identification of extracellular ligands and/or interactors is required to further understand LMK1 function in responses to C/N-nutrient stress. Further detailed genetic and physiological analyses of this protein may help to clarify its function and the as yet undetermined physiological processes controlled by C/N-nutrient availability.

## Data Availability Statement

The datasets generated for this study can be found in the ProteomeXchange (PXD016507), jPOST (JPST000703).

## Author Contributions

XL, MSa, HN, and TS designed the experiments. XL, MSa, YL, YN, SS, SY, RF, JL, and TS performed the experiments. XL, MSa, MSt, JL, HN, and TS analyzed the data. XL, MSa, HN, TS, and JY wrote the article. YS, WS, MSt, and JL edited the article. All authors commented on and approved the manuscript.

## Conflict of Interest

The authors declare that the research was conducted in the absence of any commercial or financial relationships that could be construed as a potential conflict of interest. The reviewer LG declared a past co-authorship with one of the authors JY to the handling Editor.

## Footnotes

1 http://www.maxquant.org/

2 ftp://ftp.arabidopsis.org/home/tair/Proteins/TAIR10_protein_lists/

3 http://www.maxquant.org/

4 https://string-db.org/

## Supplementary Material

FIGURE S1 Signal peptide prediction and alignment of the kinase domains of members of the LRR-RLK class VIII-2 subfamily. **(A)** Predicted signal peptide region of LMK1 protein, as determined by the SignalP-4.1 servers (http://www.cbs.dtu.dk/services/SignalP/). **(B)** Alignment of the kinase domains of members of the LRR-RLK class VIII-2 subfamily. The arrowhead indicates the position of the conserved Asp residue (LMK1D805), which is essential for kinase activity in these proteins.

FIGURE S2 Subcellular localization of LMK1 protein. **(A)** Confocal laser microscopy showing the subcellular localization of LMK1-GFP transiently expressed in *N. benthamiana* leaves. GFP was the control for fluorescent protein. Confocal microscopic images were taken 48 h after transfection. DIC, differential interference contrast image. **(B)** Immunoblotting analysis using anti-GFP antibody of water-soluble (supernatant: S) and insoluble membrane (pellet: P) fractions of lysate from N. benthamiana leaves expressing GFP or LMK1-GFP protein. Protein extraction buffer contained no detergent. Closed and open arrowheads indicate the position of GFP and LMK1-GFP, respectively.

FIGURE S3 Expression check of LMK1 proteins in *N. benthamiana* leaves. Intact LMK1 and mutated LMK1 proteins fused with GFP were transiently overexpressed in *N. benthamiana* leaves. Expression of each construct was confirmed by confocal microscopic analysis. Photos were taken 48 h after infiltration. Mock, mock treatment by infection of *Agrobacterium* carrying the p19 vector alone.

TABLE S1 Primers used in this study.

TABLE S2 List of C/N-nutrient responsive phosphoproteins identified by LC-MS/MS analysis.

## References

[B1] Aguilar-Hernández V. Aguilar-Henonin L. Guzmán P. (2011). Diversity in the architecture of ATLs, a family of plant ubiquitin-ligases, leads to recognition and targeting of substrates in different cellular environments. *PLoS One* 6:e23934. 10.1371/journal.pone.0023934 21887349 PMC3161093

[B2] Alers S. Wesselborg S. Stork B. (2014). ATG13: Just a companion, or an executor of the autophagic program? *Autophagy* 10 944–956. 10.4161/auto.28987 24879146 PMC4091178

[B3] Aoyama S. Huarancca Reyes T. Guglielminetti L. Lu Y. Morita Y. Sato T. (2014). Ubiquitin ligase ATL31 functions in leaf senescence in response to the balance between atmospheric CO_2_ and nitrogen availability in arabidopsis. *Plant Cell Physiol.* 55 293–305. 10.1093/pcp/pcu002 24399238 PMC3913444

[B4] Baena-González E. Hanson J. (2017). Shaping plant development through the SnRK1-TOR metabolic regulators. *Curr. Opin. Plant Biol.* 35 152–157. 10.1016/j.pbi.2016.12.004 28027512

[B5] Baena-González E. Rolland F. Thevelein J. M. Sheen J. (2007). A central integrator of transcription networks in plant stress and energy signalling. *Nature* 448 938–942. 10.1038/nature06069 17671505

[B6] Chevalier D. Morris E. R. Walker J. C. (2009). 14-3-3 and FHA Domains Mediate Phosphoprotein Interactions. *Annu. Rev. Plant Biol.* 60 67–91. 10.1146/annurev.arplant.59.032607.092844 19575580

[B7] Cho Y. H. Hong J. W. Kim E. C. Yoo S. D. (2012). Regulatory functions of SnRK1 in stress-responsive gene expression and in plant growth and development. *Plant Physiol.* 158 1955–1964. 10.1104/pp.111.189829 22232383 PMC3320198

[B8] Chollet R. Vidal J. O’Leary M. H. (1996). Phosphoenolpyruvate carboxylase: A ubiquitous, highly regulated enzyme in plants. *Annu. Rev. Plant Physiol. Plant Mol. Biol.* 47 273–298. 10.1146/annurev.arplant.47.1.273 15012290

[B9] Choudhary M. K. Nomura Y. Wang L. Nakagami H. Somers D. E. (2015). Quantitative Circadian Phosphoproteomic Analysis of Arabidopsis Reveals Extensive Clock Control of Key Components in Physiological, Metabolic, and Signaling Pathways. *Mol Cell Proteomics.* 14 2243–2260. 10.1074/mcp.M114.047183 26091701 PMC4528250

[B10] Coll N. S. Epple P. Dangl J. L. (2011). Programmed cell death in the plant immune system. *Cell Death Differ.* 18 1247–1256. 10.1038/cdd.2011.37 21475301 PMC3172094

[B11] Comparot S. Lingiah G. Martin T. (2003). Function and specificity of 14-3-3 proteins in the regulation of carbohydrate and nitrogen metabolism. *J. Exp. Bot.* 54 595–604. 10.1093/jxb/erg057 12508070

[B12] Coruzzi G. M. Zhou L. (2001). Carbon and nitrogen sensing and signaling in plants: Emerging “matrix effects.” *Curr. Opin. Plant Biol.* 4 247–253. 10.1016/S1369-5266(00)00168-0 11312136

[B13] Cox J. Mann M. (2008). MaxQuant enables high peptide identification rates, individualized p.p.b.-range mass accuracies and proteome-wide protein quantification. *Nat. Biotechnol.* 26 1367–1372. 10.1038/nbt.1511 19029910

[B14] Curtis M. D. Grossniklaus U. (2003). A Gateway Cloning Vector Set for High-Throughput Functional Analysis of Genes in Planta. *Plant Physiol.* 133 462–469. 10.1104/pp.103.027979 14555774 PMC523872

[B15] Dai C. Lee Y. Lee I. C. Nam H. G. Kwak J. M. (2018). Calmodulin 1 regulates senescence and ABA response in Arabidopsis. *Front. Plant Sci.* 9:1–13. 10.3389/fpls.2018.00803 30013580 PMC6036150

[B16] Diévart A. Clark S. E. (2004). LRR-containing receptors regulating plant development and defense. *Development* 131 251–261. 10.1242/dev.00998 14701679

[B17] Du C. Li X. Chen J. Chen W. Li B. Li C. (2016). Receptor kinase complex transmits RALF peptide signal to inhibit root growth in Arabidopsis. *Proc. Natl. Acad. Sci. U. S. A.* 113 E8326–E8334. 10.1073/pnas.1609626113 27930296 PMC5187724

[B18] Dünser K. Gupta S. Herger A. Feraru M. I. Ringli C. Kleine−Vehn J. (2019). Extracellular matrix sensing by FERONIA and Leucine−Rich Repeat Extensins controls vacuolar expansion during cellular elongation in Arabidopsis thaliana. *EMBO J* 38 1–12. 10.15252/embj.2018100353 30850388 PMC6443208

[B19] Emanuelle S. Doblin M. S. Stapleton D. I. Bacic A. Gooley P. R. (2016). Molecular Insights into the Enigmatic Metabolic Regulator. *SnRK1. Trends Plant Sci.* 21 341–353. 10.1016/j.tplants.2015.11.001 26642889

[B20] Feng W. Kita D. Peaucelle A. Cartwright H. N. Doan V. Duan Q. (2018). The FERONIA Receptor Kinase Maintains Cell-Wall Integrity during Salt Stress through Ca^2 +^ Signaling. *Curr. Biol.* 28 666.e–675.e. 10.1016/j.cub.2018.01.023 666–675.e5, 29456142 PMC5894116

[B21] Figueroa C. M. Feil R. Ishihara H. Watanabe M. Kölling K. Krause U. (2016). Trehalose 6-phosphate coordinates organic and amino acid metabolism with carbon availability. *Plant J.* 85 410–423. 10.1111/tpj.13114 26714615

[B22] Figueroa C. M. Lunn J. E. (2016). A Tale of Two Sugars: Trehalose 6-Phosphate and Sucrose. *Plant Physiol.* 172 7–27. 10.1104/pp.16.00417 27482078 PMC5074632

[B23] Haruta M. Burch H. L. Nelson R. B. Barrett-Wilt G. Kline K. G. Mohsin S. B. (2010). Molecular characterization of mutant Arabidopsis plants with reduced plasma membrane proton pump activity. *J. Biol. Chem.* 285 17918–17929. 10.1074/jbc.M110.101733 20348108 PMC2878554

[B24] Haruta M. Sabat G. Stecker K. Minkoff B. B. Sussman M. R. (2014). A peptide hormone and its receptor protein kinase regulate plant cell expansion. *Science (80-.).* 343 408–411. 10.1126/science.1244454 24458638 PMC4672726

[B25] Huarancca Reyes T. Maekawa S. Sato T. Yamaguchi J. (2015). The Arabidopsis ubiquitin ligase ATL31 is transcriptionally controlled by WRKY33 transcription factor in response to pathogen attack. *Plant Biotechnol.* 32 11–19. 10.5511/plantbiotechnology.14.1201b

[B26] Huarancca Reyes T. Scartazza A. Pompeiano A. Ciurli A. Lu Y. Guglielminetti L. (2018). Nitrate reductase modulation in response to changes in C/N balance and nitrogen source in Arabidopsis. *Plant Cell Physiol.* 59 1248–1254. 10.1093/pcp/pcy065 29860377

[B27] Ishihama Y. Rappsilber J. Andersen J. S. Mann M. (2002). Microcolumns with self-assembled particle frits for proteomics. *J. Chromatogr.* 6 979 233–239. 10.1016/s0021-9673(02)01402-4 12498253

[B28] Izui K. Matsumura H. Furumoto T. Kai Y. (2004). PHOSPHO ENOL PYRUVATE CARBOXYLASE: A New Era of Structural Biology. *Annu. Rev. Plant Biol.* 55 69–84. 10.1146/annurev.arplant.55.031903.141619 15725057

[B29] Jaspert N. Throm C. Oecking C. (2011). Arabidopsis 14-3-3 proteins: Fascinating and less fascinating aspects. *Front. Plant Sci.* 2:1–8. 10.3389/fpls.2011.00096 22639620 PMC3355631

[B30] Kadota Y. Sklenar J. Derbyshire P. Stransfeld L. Asai S. Ntoukakis V. (2014). Direct Regulation of the NADPH Oxidase RBOHD by the PRR-Associated Kinase BIK1 during Plant Immunity. *Mol. Cell* 54 43–55. 10.1016/j.molcel.2014.02.021 24630626

[B31] Kinoshita T. Shimazaki K. I. (1999). Blue light activates the plasma membrane H^+^-ATPase by phosphorylation of the C-terminus in stomatal guard cells. *EMBO J.* 18 5548–5558. 10.1093/emboj/18.20.5548 10523299 PMC1171623

[B32] Koyama T. Furutani M. Tasaka M. Ohme-Takagi M. (2007). TCP transcription factors control the morphology of shoot lateral organs via negative regulation of the expression of boundary-specific genes in Arabidopsis. *Plant Cell* 19 473–484. 10.1105/tpc.106.044792 17307931 PMC1867346

[B33] Kwak J. M. Mori I. C. Pei Z. M. Leonhard N. Angel Torres M. Dangl J. L. (2003). NADPH oxidase AtrbohD and AtrbohF genes function in ROS-dependent ABA signaling in arabidopsis. *EMBO J.* 22 2623–2633. 10.1093/emboj/cdg277 12773379 PMC156772

[B34] Le M. H. Cao Y. Zhang X. C. Stacey G. (2014). LIK1, a CERK1-interacting kinase, regulates plant immune responses in arabidopsis. *PLoS One* 9:1–10. 10.1371/journal.pone.0102245 25036661 PMC4103824

[B35] Liao H. Tang R. Zhang X. Luan S. Yu F. (2017). FERONIA Receptor Kinase at the Crossroads of Hormone Signaling and Stress Responses. *Plant Cell Physiol.* 58 1143–1150. 10.1093/pcp/pcx048 28444222

[B36] Lim P. O. Kim H. J. Nam H. G. (2007). Leaf Senescence. *Annu. Rev. Plant Biol.* 58 115–136. 10.1146/annurev.arplant.57.032905.105316 17177638

[B37] Lu Y. Sasaki Y. Li X. Mori I. C. Matsuura T. Hirayama T. (2015). ABI1 regulates carbon/nitrogen-nutrient signal transduction independent of ABA biosynthesis and canonical ABA signalling pathways in Arabidopsis. *J. Exp. Bot.* 66 2763–2771. 10.1093/jxb/erv086 25795738 PMC4986877

[B38] Lunn J. E. Feil R. Hendriks J. H. M. Gibon Y. Morcuende R. Osuna D. (2006). Sugar-induced increases in trehalose 6-phosphate are correlated with redox activation of ADPglucose pyrophosphorylase and higher rates of starch synthesis in Arabidopsis thaliana. *Biochem. J.* 397 139–148. 10.1042/BJ20060083 16551270 PMC1479759

[B39] Macho A. P. Zipfel C. (2014). Plant PRRs and the activation of innate immune signaling. *Mol. Cell* 54 263–272. 10.1016/j.molcel.2014.03.028 24766890

[B40] Mackintosh C. (2004). Dynamic interactions between 14-3-3 proteins and phosphoproteins regulate diverse cellular processes. *Biochem. J.* 381 329–342. 10.1042/BJ20031332 15167810 PMC1133837

[B41] Maekawa S. Sato T. Asada Y. Yasuda S. Yoshida M. Chiba Y. (2012). The Arabidopsis ubiquitin ligases ATL31 and ATL6 control the defense response as well as the carbon/nitrogen response. *Plant Mol. Biol.* 79 217–227. 10.1007/s11103-012-9907-0 22481162

[B42] Mair A. Pedrotti L. Wurzinger B. Anrather D. Simeunovic A. Weiste C. (2015). SnRK1-triggered switch of bZIP63 dimerization mediates the low-energy response in plants. *Elife* 4 1–33. 10.7554/eLife.05828 26263501 PMC4558565

[B43] Martin T. Oswald O. Graham I. (2002). Arabidopsis seedling growth, storage lipid mobilization, and photosynthetic gene expression are regulated by carbon: nitrogen availability. *Plant Physiol.* 128 472–481. 10.1104/pp.010475 11842151 PMC148910

[B44] Mizushima N. (2010). The role of the Atg1/ULK1 complex in autophagy regulation. *Curr. Opin. Cell Biol.* 22 132–139. 10.1016/j.ceb.2009.12.004 20056399

[B45] Mukhtar M. S. McCormack M. E. Argueso C. T. Pajerowska-Mukhtar K. M. (2016). Pathogen Tactics to Manipulate Plant Cell Death. *Curr. Biol.* 26 R608–R619. 10.1016/j.cub.2016.02.051 27404256

[B46] Nakagami H. (2014). StageTip-based HAMMOC, an efficient and inexpensive phosphopeptide enrichment method for plant shotgun phosphoproteomics. *Methods Mol. Biol.* 1072 595–607. 10.1007/978-1-62703-631-3_40 24136549

[B47] Nakagawa T. Kurose T. Hino T. Tanaka K. Kawamukai M. Niwa Y. (2007). Development of series of gateway binary vectors, pGWBs, for realizing efficient construction of fusion genes for plant transformation. *J. Biosci. Bioeng.* 104 34–41. 10.1263/jbb.104.34 17697981

[B48] Nicolas M. Cubas P. (2016). TCP factors: New kids on the signaling block. *Curr. Opin. Plant Biol.* 33 33–41. 10.1016/j.pbi.2016.05.006 27310029

[B49] Okuda S. Watanabe Y. Moriya Y. Kawano S. Yamamoto T. Matsumoto M. (2017). JPOSTrepo: An international standard data repository for proteomes. *Nucleic Acids Res.* 45 D1107–D1111. 10.1093/nar/gkw1080 27899654 PMC5210561

[B50] Okumura M. Inoue S. I. Kuwata K. Kinoshita T. (2016). Photosynthesis activates plasma membrane H^+^-ATPase via sugar accumulation. *Plant Physiol.* 171 580–589. 10.1104/pp.16.00355 27016447 PMC4854722

[B51] O’Leary B. Park J. Plaxton W. C. (2011). The remarkable diversity of plant PEPC (phosphoenolpyruvate carboxylase): recent insights into the physiological functions and post-translational controls of non-photosynthetic PEPCs. *Biochem. J.* 436 15–34. 10.1042/BJ20110078 21524275

[B52] Olsen J. V. de Godoy L. M. Li G. Macek B. Mortensen P. Pesch R. (2005). Parts per million mass accuracy on an Orbitrap mass spectrometer via lock mass injection into a C-trap. *Mol. Cell Proteomics.* 4 2010–2021. 10.1074/mcp.T500030-MCP200 16249172

[B53] Palatnik J. F. Allen E. Wu X. Schommer C. Schwab R. Carrington J. C. (2003). Control of leaf morphogenesis by microRNAs. *Nature* 425 257–263. 10.1038/nature01958 12931144

[B54] Paul M. J. Primavesi L. F. Jhurreea D. Zhang Y. (2008). Trehalose metabolism and signaling. *Annu. Rev. Plant Biol.* 59, 417–441. 10.1146/annurev.arplant.59.032607.092945 18257709

[B55] Palmgren M. G. (2001). PLANT PLASMA MEMBRANE H^+^-ATPases: Powerhouses for Nutrient Uptake. *Annu. Rev. Plant Physiol. Plant Mol. Biol.* 52 817–845. 10.1146/annurev.arplant.52.1.817 11337417

[B56] Pedrotti L. Weiste C. Nägele T. Wolf E. Lorenzin F. Dietrich K. (2018). Snf1-RELATED KINASE1-Controlled C/S_1_-bZIP Signaling Activates Alternative Mitochondrial Metabolic Pathways to Ensure Plant Survival in Extended Darkness. *Plant Cell* 30 495–509. 10.1105/tpc.17.00414 29348240 PMC5868691

[B57] Ramon M. Dang T. V. T. Broeckx T. Hulsmans S. Crepin N. Sheen J. (2019). Default activation and nuclear translocation of the plant cellular energy sensor SnRK1 regulate metabolic stress responses and development. *Plant Cell* 31 1614–1632. 10.1105/tpc.18.00500 31123051 PMC6635846

[B58] Rolland F. Baena-González E. Sheen J. (2006). SUGAR SENSING AND SIGNALING IN PLANTS: Conserved and Novel Mechanisms. *Annu. Rev. Plant Biol.* 57 675–709. 10.1146/annurev.arplant.57.032905.105441 16669778

[B59] Sanagi M. Lu Y. Aoyama S. Morita Y. Mitsuda N. Ikeda M. (2018). Sugar-responsive transcription factor bZIP3 affects leaf shape in Arabidopsis plants. *Plant Biotechnol.* 35 167–170. 10.5511/plantbiotechnology.18.0410a 31819719 PMC6879397

[B60] Sato T. Maekawa S. Yasuda S. Domeki Y. Sueyoshi K. Fujiwara M. (2011). Identification of 14-3-3 proteins as a target of ATL31 ubiquitin ligase, a regulator of the C/N response in Arabidopsis. *Plant J* 68 137–146. 10.1111/j.1365-313X.2011.04673.x 21668537

[B61] Sato T. Maekawa S. Yasuda S. Sonoda Y. Katoh E. Ichikawa T. (2009). CNI1/ATL31, a RING-type ubiquitin ligase that functions in the carbon/nitrogen response for growth phase transition in Arabidopsis seedlings. *Plant J.* 60 852–864. 10.1111/j.1365-313X.2009.04006.x 19702666

[B62] Schallus T. Jaeckh C. Fehér K. Palma A. S. Liu Y. Simpson J. C. (2008). Malectin: a novel carbohydrate-binding protein of the endoplasmic reticulum and a candidate player in the early steps of protein *N*-glycosylation. *Mol. Biol. Cell* 19, 3404–3414. 10.1091/mbc.E08-04-0354 18524852 PMC2488313

[B63] Schommer C. Palatnik J. F. Aggarwal P. Chételat A. Cubas P. Farmer E. E. (2008). Control of jasmonate biosynthesis and senescence by miR319 targets. *PLoS Biol.* 6:1991–2001. 10.1371/journal.pbio.0060230 18816164 PMC2553836

[B64] Schroeder M. J. Shabanowitz J. Schwartz J. C. Hunt D. F. Coon J. (2004). A neutral loss activation method for improved phosphopeptide sequence analysis by quadrupole ion trap mass spectrometry. *J. Anal Chem.* 76 3590–3598. 10.1021/ac0497104 15228329

[B65] Serrano M. Parra S. Alcaraz L. D. Guzmán P. (2006). The ATL gene family from Arabidopsis thaliana and Oryza sativa comprises a large number of putative ubiquitin ligases of the RING-H2 type. *J. Mol. Evol.* 62 434–445. 10.1007/s00239-005-0038-y 16557337

[B66] Shi J. Yi K. Liu Y. Xie L. Zhou Z. Chen Y. (2015). Phosphoenolpyruvate Carboxylase in Arabidopsis Leaves Plays a Crucial Role in Carbon and Nitrogen Metabolism. *Plant Physiol.* 167 671–681. 10.1104/pp.114.254474 25588735 PMC4348777

[B67] Shiu S. H. Bleecker A. B. (2003). Expansion of the receptor-like kinase/Pelle gene family and receptor-like proteins in Arabidopsis. *Plant Physiol.* 132 530–543. 10.1104/pp.103.021964 12805585 PMC166995

[B68] Smeekens S. Ma J. Hanson J. Rolland F. (2010). Sugar signals and molecular networks controlling plant growth. *Curr. Opin. Plant Biol.* 13 274–279. 10.1016/j.pbi.2009.12.002 20056477

[B69] Stegmann M. Monaghan J. Smakowska-Luzan E. Rovenich H. Lehner A. Holton N. (2017). The receptor kinase FER is a RALF-regulated scaffold controlling plant immune signaling. *Science* 355 287–289. 10.1126/science.aal2541 28104890

[B70] Suttangkakul A. Li F. Chung T. Vierstra R. D. (2011). The ATG1/ATG13 Protein Kinase Complex Is Both a Regulator and a Target of Autophagic Recycling in Arabidopsis. *Plant Cell* 23 3761–3779. 10.1105/tpc.111.090993 21984698 PMC3229148

[B71] Suzuki K. Ohsumi Y. (2010). Current knowledge of the pre-autophagosomal structure (PAS). *FEBS Lett.* 584 1280–1286. 10.1016/j.febslet.2010.02.001 20138172

[B72] Svennelid F. Olsson A. Piotrowski M. Rosenquist M. Ottman C. Larsson C. (1999). Phosphorylation of Thr-948 at the C terminus of the plasma membrane H^+^-ATPase creates a binding site for the regulatory 14-3-3 protein. *Plant Cell* 11 2379–2391. 10.1105/tpc.11.12.2379 10590165 PMC144135

[B73] Taylor L. Nunes-Nesi A. Parsley K. Leiss A. Leach G. Coates S. (2010). Cytosolic pyruvate,orthophosphate dikinase functions in nitrogen remobilization during leaf senescence and limits individual seed growth and nitrogen content. *Plant J.* 62 641–652. 10.1111/j.1365-313X.2010.04179.x 20202167

[B74] Torres M. A. Dangl J. L. (2005). Functions of the respiratory burst oxidase in biotic interactions, abiotic stress and development. *Curr. Opin. Plant Biol.* 8 397–403. 10.1016/j.pbi.2005.05.014 15939662

[B75] Torres M. A. Dangl J. L. Jones J. D. G. (2002). Arabidopsis gp91^phox^ homologues Atrbohd and Atrbohf are required for accumulation of reactive oxygen intermediates in the plant defense response. *Proc. Natl. Acad. Sci. U. S. A.* 99 517–522. 10.1073/pnas.012452499 11756663 PMC117592

[B76] Tyanova S. Temu T. Cox J. (2016a). The MaxQuant computational platform for mass spectrometry-based shotgun proteomics. *Nat. Protoc.* 11 2301–2319. 10.1038/nprot.2016.136 27809316

[B77] Tyanova S. Temu T. Sinitcyn P. Carlson A. Hein M. Y. Geiger T. (2016b). The Perseus computational platform for comprehensive analysis of (prote)omics data. *Nat. Methods.* 13 731–740. 10.1038/nmeth.3901 27348712

[B78] Vidal E. A. Gutiérrez R. A. (2008). A systems view of nitrogen nutrient and metabolite responses in Arabidopsis. *Curr. Opin. Plant Biol.* 11 521–529. 10.1016/j.pbi.2008.07.003 18775665

[B79] von Mering C. Jensen L. J. Snel B. Hooper S. D. Krupp M. Foglierini M. (2005). STRING: Known and predicted protein-protein associations, integrated and transferred across organisms. *Nucleic Acids Res.* 33 433–437. 10.1093/nar/gki005 15608232 PMC539959

[B80] Wahl V. Ponnu J. Schlereth A. Arrivault S. Langenecker T. Franke A. (2013). Regulation of flowering by trehalose-6-phosphate signaling in *Arabidopsis thaliana*. *Science* 339 704–707. 10.1126/science.1230406 23393265

[B81] Wingler A. Delatte T. L. O’Hara L. E. Primavesi L. F. Jhurreea D. Paul M. J. (2012). Trehalose 6-Phosphate Is Required for the Onset of Leaf Senescence Associated with High Carbon Availability. *Plant Physiol.* 158 1241–1251. 10.1104/pp.111.191908 22247267 PMC3291265

[B82] Xu G. Chen W. Song L. Chen Q. Zhang H. Liao H. (2019). FERONIA phosphorylates E3 ubiquitin ligase ATL6 to modulate the stability of 14-3-3 proteins in plant C/N responses. *J. Exp. Bot.* 70 6375–6388. 10.1093/jxb/erz378 31433471 PMC6859809

[B83] Yang L. Ye C. Zhao Y. Cheng X. Wang Y. Jiang Y. Q. (2018). An oilseed rape WRKY-type transcription factor regulates ROS accumulation and leaf senescence in Nicotiana benthamiana and Arabidopsis through modulating transcription of RbohD and RbohF. *Planta* 247 1323–1338. 10.1007/s00425-018-2868-z 29511814

[B84] Yasuda S. Aoyama S. Hasegawa Y. Sato T. Yamaguchi J. (2017). Arabidopsis CBL-Interacting Protein Kinases Regulate Carbon/Nitrogen-Nutrient Response by Phosphorylating Ubiquitin Ligase ATL31. *Mol. Plant* 10 605–618. 10.1016/j.molp.2017.01.005 28111287

[B85] Yasuda S. Sato T. Maekawa S. Aoyama S. Fukao Y. Yamaguchi J. (2014). Phosphorylation of arabidopsis ubiquitin ligase ATL31 is critical for plant carbon/nitrogen nutrient balance response and controls the stability of 14-3-3 proteins. *J. Biol. Chem.* 289 15179–15193. 10.1074/jbc.M113.533133 24722992 PMC4140878

[B86] Yoo S. D. Cho Y. H. Sheen J. (2007). Arabidopsis mesophyll protoplasts: A versatile cell system for transient gene expression analysis. *Nat. Protoc.* 2 1565–1572. 10.1038/nprot.2007.199 17585298

[B87] Zhai Z. Keereetaweep J. Liu H. Feil R. Lunn J. E. Shanklin J. (2018). Trehalose 6-phosphate positively regulates fatty acid synthesis by stabilizing WRINKLED1. *Plant Cell* 30 2616–2627. 10.1105/tpc.18.00521 30249634 PMC6241258

[B88] Zhang Y. Primavesi L. F. Jhurreea D. Andralojc P. J. Mitchell R. A. C. Powers S. J. (2009). Inhibition of SNF1-related protein kinasel activity and regulation of metabolic pathways by trehalose-6-phosphate. *Plant Physiol.* 149 1860–1871. 10.1104/pp.108.133934 19193861 PMC2663748

