# Supplementary material for: Protein Phosphorylation Dynamics Under Carbon/Nitrogen-Nutrient Stress and Identification of a Cell Death-Related Receptor-Like Kinase in Arabidopsis
Source: Front Plant Sci. 2020 Apr 3;11:377. doi: 10.3389/fpls.2020.00377 (PMC7145971; doi:10.3389/fpls.2020.00377)
Supplement: FIGURE S1 — Signal peptide prediction and alignment of the kinase domains of members of the LRR-RLK class VIII-2 subfamily. (A) Predicted signal peptide region of LMK1 protein, as determined by the SignalP-4.1 servers (http://www.cbs.dtu.dk/services/SignalP/). (B) Alignment of the kinase domains of members of the LRR-RLK class VIII-2 subfamily. The arrowhead indicates the position of the conserved Asp residue (LMK1D805), which is essential for kinase activity in these proteins. [file Data_Sheet_1.pdf]

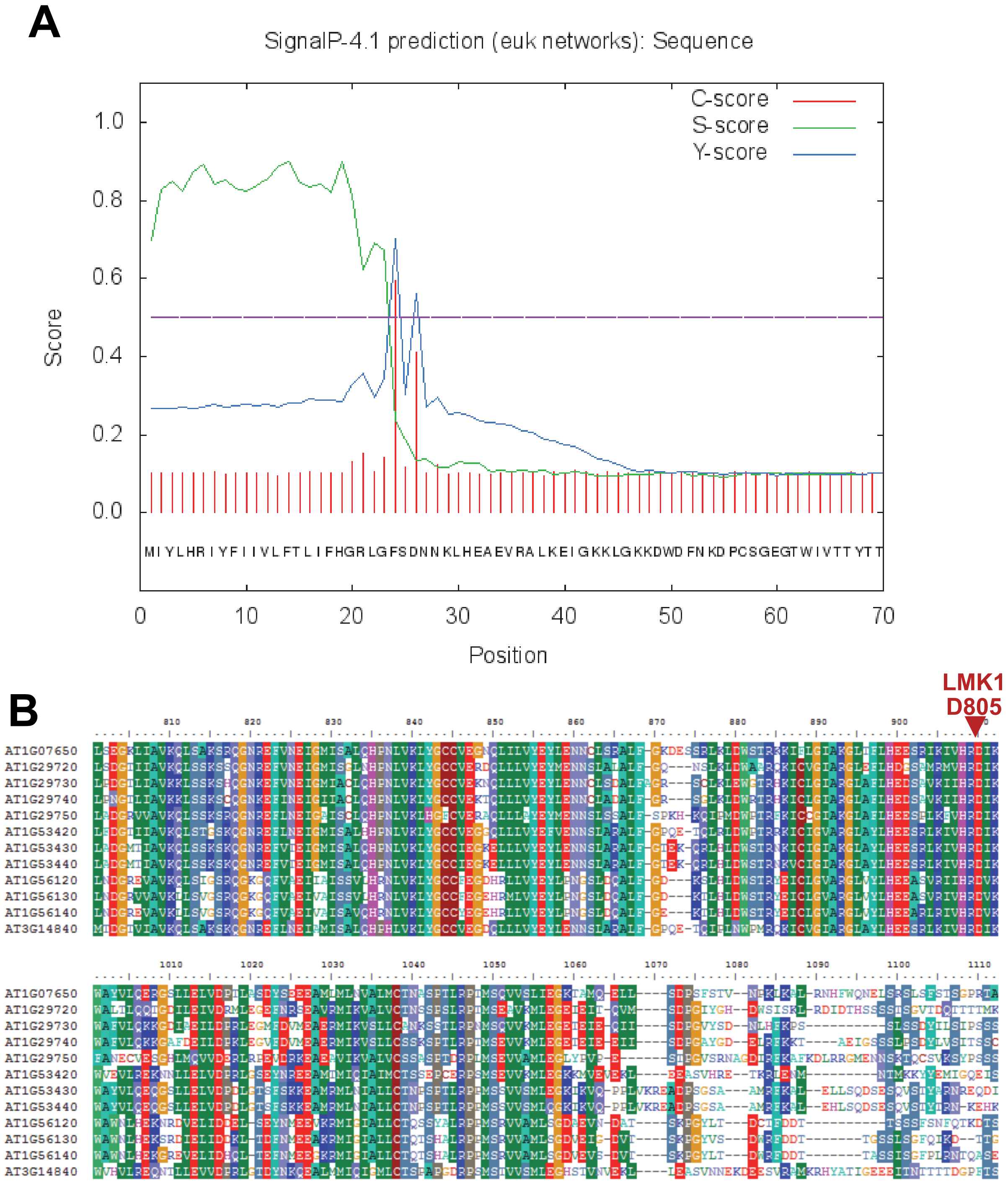

Supplementary Figure S1. Signal peptide prediction and alignment of the kinase domains of members of the LRR-RLK class VIII-2 subfamily.

(A) Predicted signal peptide region of LMK1 protein, as determined by the SignalP-4.1 servers (http://www.cbs.dtu.dk/services/SignalP/).

(B) Alignment of the kinase domains of members of the LRR-RLK class VIII-2 subfamily. The arrowhead indicates the position of the conserved Asp residue (LMK1D805), which is essential for kinase activity in these proteins.

Supplemental Fig. S1

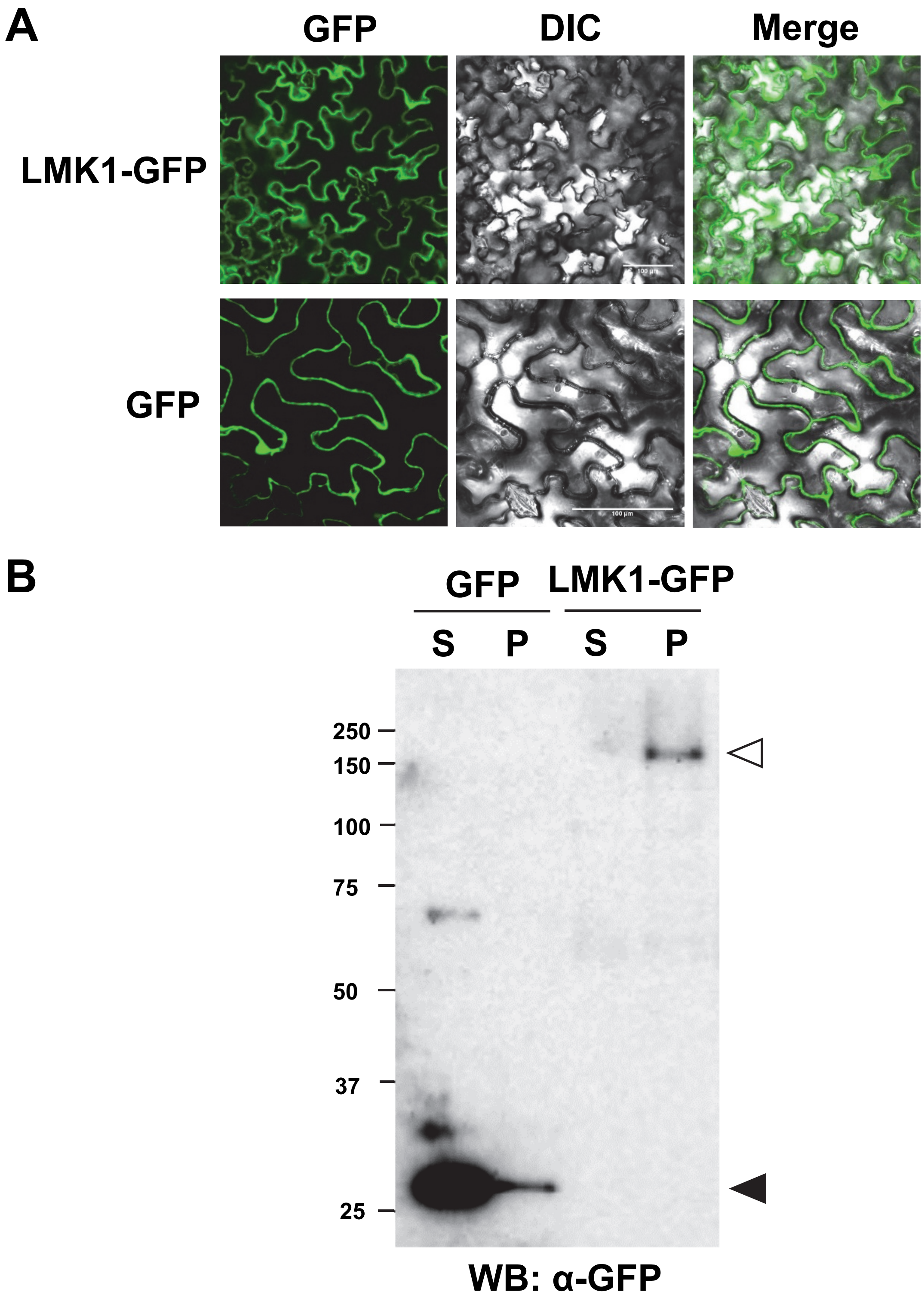

Supplementary Figure S2. Subcellular localization of LMK1 protein.

(A) Confocal laser microscopy showing the subcellular localization of LMK1-GFP transiently expressed in *N. benthamiana* leaves. GFP was the control for fluorescent protein. Confocal microscopic images were taken 48 h after transfection. DIC, differential interference contrast image.

(B) Immunoblotting analysis using anti-GFP antibody of water-soluble (supernatant: S) and insoluble membrane (pellet: P) fractions of lysate from *N. benthamiana* leaves expressing GFP or LMK1-GFP protein. Protein extraction buffer contained no detergent. Closed and open arrowheads indicate the position of GFP and LMK1-GFP, respectively.

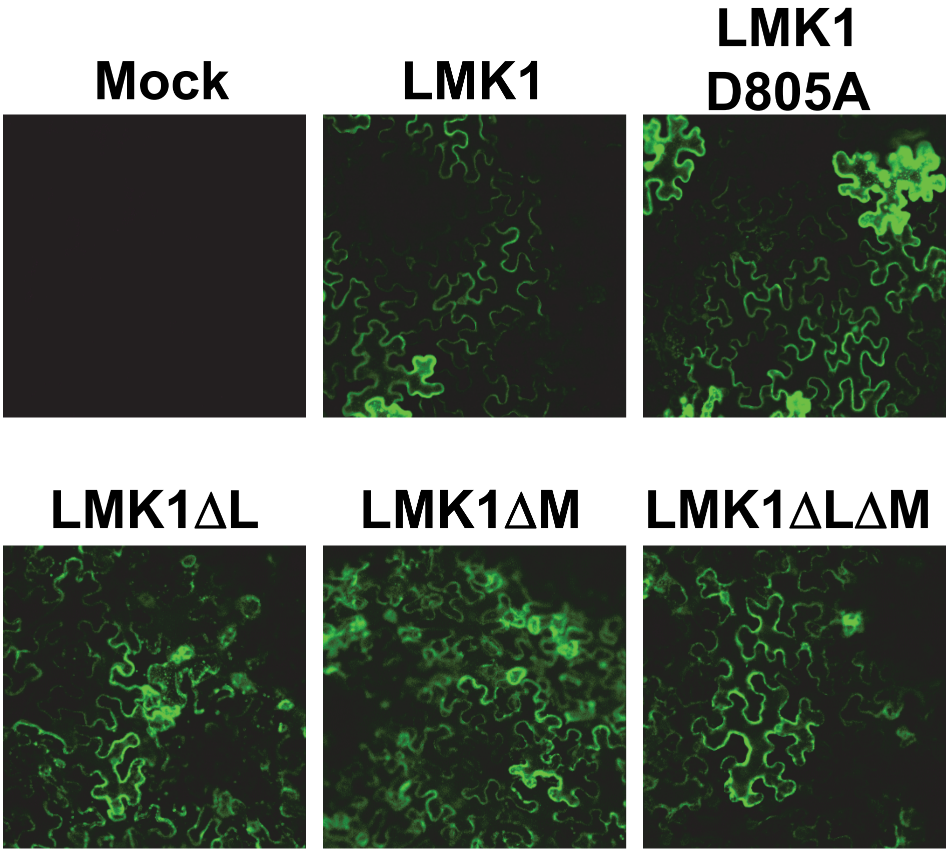

Supplementary Figure S3. Expression check of LMK1 protein constructs in *N. benthamiana* leaves.

Intact LMK1 and mutated LMK1 proteins fused with GFP were transiently overexpressed in *N. benthamiana* leaves. Expression of each construct was confirmed by confocal microscopic analysis. Photos were taken 48 h after infiltration. Mock, mock treatment by infection of Agrobacterium carrying the p19 vector alone.
