# Supplementary material for: Protein Phosphorylation Dynamics Under Carbon/Nitrogen-Nutrient Stress and Identification of a Cell Death-Related Receptor-Like Kinase in Arabidopsis
Source: Front Plant Sci. 2020 Apr 3;11:377. doi: 10.3389/fpls.2020.00377 (PMC7145971; doi:10.3389/fpls.2020.00377)
Supplement: TABLE S1 — Primers used in this study. [file Table_1.pdf]

Supplementary Table S1 Primers used in this study.

Primer	Sequence (Forward)	Sequence (Reverse)
**Plasmid construction**		
LMK1 pENTR/D-TOPO	CACCATGATATATTTACATCGCATA	ATCCAAAAGTCCGGTTTTCTC
LMK1D805A	GTACACAGAGCTATAAAGGCA	TGCCTTTATAGCTCTGTGTAC
LMK1ΔL	TCTAAGCTTCGTAAATTGTACATA	TATGTACAATTTACGAAGCTTAGA
LMK1ΔM	TATCATTTATATTCTGTGGAACCC	GGGTTCCACAGAATATAAATGATA
LMK1ΔLΔM	TCTAAGCTTCGTTCTGTGGAACCC	GGGTTCCACAGAACGAAGCTTAGA
**qRT-PCR**		
CIPK7	AAAATCTCCCGCTTCATTGTC	GTAGTGATCAAAGCTTATGGAATCG
CIPK12	AAAGCTGCTTACGGTTGGTG	TCAATTTCTCTAACGATCCTCTCC
CIPK14	CTAACGGACGAGCTGGTTATG	GACGAAGAAAGGCCTGAGTG
RBCS1A	CTTCCCTTGTTCGGTTGCA	TGCACTCTTCCACTTCCTTCAA
SnRK1.1	ATGAAGTGCAGATGGGTTCC	ACATTGGGCGACTTAACAGC
CHS	AAGCGCATGTGCGACAAG	TCCTCCGTCAGATGCATGTG
18S rRNA	CGGCTACCACATCCAAGGAA	AGCCGCGGTAATTCCAGC
